# A physical map of traits of agronomic importance based on potato and tomato genome sequences

**DOI:** 10.3389/fgene.2023.1197206

**Published:** 2023-07-25

**Authors:** Christiane Gebhardt

**Affiliations:** Max Planck Institute for Plant Breeding Research, Cologne, Germany

**Keywords:** potato, tomato, pepper, physical map, pathogen resistance, yield, sugar content, maturity

## Abstract

Potato, tomato, pepper, and eggplant are worldwide important crop and vegetable species of the Solanaceae family. Molecular linkage maps of these plants have been constructed and used to map qualitative and quantitative traits of agronomic importance. This research has been undertaken with the vision to identify the molecular basis of agronomic characters on the one hand, and on the other hand, to assist the selection of improved varieties in breeding programs by providing DNA-based markers that are diagnostic for specific agronomic characters. Since 2011, whole genome sequences of tomato and potato became available in public databases. They were used to combine the results of several hundred mapping and map-based cloning studies of phenotypic characters between 1988 and 2022 in physical maps of the twelve tomato and potato chromosomes. The traits evaluated were qualitative and quantitative resistance to pathogenic oomycetes, fungi, bacteria, viruses, nematodes, and insects. Furthermore, quantitative trait loci for yield and sugar content of tomato fruits and potato tubers and maturity or earliness were physically mapped. Cloned genes for pathogen resistance, a few genes underlying quantitative trait loci for yield, sugar content, and maturity, and several hundred candidate genes for these traits were included in the physical maps. The comparison between the physical chromosome maps revealed, in addition to known intrachromosomal inversions, several additional inversions and translocations between the otherwise highly collinear tomato and potato genomes. The integration of the positional information from independent mapping studies revealed the colocalization of qualitative and quantitative loci for resistance to different types of pathogens, called resistance hotspots, suggesting a similar molecular basis. Synteny between potato and tomato with respect to genomic positions of quantitative trait loci was frequently observed, indicating eventual similarity between the underlying genes.

## 1 Introduction

The Solanaceae are a highly diverse family of land plants. It comprises 3,000 to 4,000 species that are organized into approximately 90 genera, the largest of which is the genus *Solanum* (Knapp et al., 2004). Some species of the Solanaceae had important roles in the history of mankind as providers of edible fruits and tubers such as tomato, potato, pepper, and eggplant, drugs such as tobacco, or ornamentals such as Petunia. Members of this family have been domesticated thousands of years ago in the Americas by pre-Columbian civilizations (potato, tomato, and pepper) and in South-East Asia (eggplant) (Daunay and Laterrot, 2008). Today, crop or vegetable species of worldwide importance are the potato (*Solanum tuberosum* Group *tuberosum*), tomato (*Solanum lycopersicum*, former *Lycopersicon esculentum*), eggplant (*Solanum melongena*), and pepper (*Capsicum* species). Besides being a crop, the tomato and its wild relatives played an important role as plant models in classical genetic research, whereas potato, pepper, and eggplant were mainly subject to breeding research aimed at crop improvement (Gebhardt, 2016).

The structure of plant genomes became accessible at the molecular level approximately 40 years ago with the advent of DNA-based markers, which allowed for the first time the construction of detailed molecular linkage maps (Helentjaris et al., 1985; Helentjaris et al., 1986; Bernatzky and Tanksley, 1986). The first type of DNA-based genetic marker was restriction fragment length polymorphism (RFLP), which detects DNA variation between individuals of the same or closely related species. The individual’s genomic DNA is digested with a restriction endonuclease; the fragments are size separated by gel electrophoresis and hybridized to a labelled DNA probe (Southern, 1975). The first molecular linkage maps of tomato, potato, pepper, and eggplant were constructed based on the segregation patterns of RFLP markers (Bonierbale et al., 1988; Gebhardt et al., 1989; Gebhardt et al., 1991; Tanksley et al., 1992; Gebhardt et al., 1994; Livingstone et al., 1999; Doganlar et al., 2002; Gebhardt et al., 2003). Based on a common set of tomato RFLP markers used for linkage mapping in tomato, potato, pepper, and eggplant, it was shown that the tomato and potato genomes are highly collinear or syntenic. The 12 chromosomes of both species corresponded to 12 linkage groups. The order of the markers was preserved between the potato and tomato maps, except for a few intrachromosomal inversions (Bonierbale et al., 1988; Tanksley et al., 1992; The Tomato Genome Consortium, 2012). A higher fragmentation in syntenic blocks (genome segments smaller than whole chromosomes sharing the same marker order in different species) and chromosome rearrangements (inversions, intra-, and inter-chromosomal translocations) was observed when comparing the maps of tomato, eggplant, and pepper (Livingstone et al., 1999; Doganlar et al., 2002).

RFLP linkage maps were the starting point for the mapping of qualitative and quantitative traits of agronomic importance, first and predominantly, in potato and tomato. The aim was, on the one hand, the development of molecular diagnostic tools for marker-assisted cultivar selection in breeding programs, and, on the other hand, preparing the ground for the map-based cloning of genes controlling agronomic traits such as pathogen resistance. After the invention of the polymerase chain reaction (PCR) (Saiki et al., 1988), RFLP markers were supplemented and eventually replaced by PCR-based markers, such as microsatellites or simple sequence repeats (SSR’s) (Broun and Tanksley, 1996; Milbourne et al., 1998), amplified fragment length polymorphism (AFLP) (Meksem et al., 1995; Vos et al., 1995), and random amplified polymorphic DNA (RAPD) (Williams et al., 1990; Klein-Lankhorst et al., 1991). For practical reasons, RFLP, AFLP, and RAPD markers linked with traits of interest were often converted into locus-specific PCR-based markers. They allowed the reliable detection of specific DNA polymorphisms in large numbers of plants, such as breeding populations (for example, Kasai et al., 2000). Subsequently, automatization and cost reduction of DNA sequencing facilitated the direct detection of point mutations (single nucleotide polymorphism, SNP) and small insertion/deletion polymorphisms by comparative sequencing of individuals of the same or closely related species (Rickert et al., 2003). The next milestones were whole genome sequences of potato (The Potato Genome Sequencing Consortium, 2011; Sharma et al., 2013; Kyriakidou et al., 2020; Freire et al., 2021; Sun et al., 2022), tomato (The Tomato Genome Consortium, 2012; The 100 Tomato Genome Sequencing Consortium, 2014) and subsequently other members of the Solanaceae (Kim et al., 2014; Kim et al., 2017; Bombarely et al., 2016; Edwards et al., 2017).

Hundreds of linkage mapping studies of qualitative and quantitative traits using DNA-based markers have been performed over the last 35 years on potato and tomato. It started with monogenic traits, such as single dominant genes for pathogen resistance (*R* genes) (Young et al., 1988; Barone et al., 1990), and was soon followed by the mapping of polygenic or quantitative traits (Paterson et al., 1988; Leonards-Schippers et al., 1994). A molecular linkage map was instrumental in the positional cloning of the tomato *Pto* gene for resistance to the bacterial pathogen *Pseudomonas syringae* (Martin et al., 1993a; Martin et al., 1993b). *Pto* was one of the first plant genes isolated for pathogen resistance and the first one of the Solanaceae family. Quantitative trait locus (QTL) mapping led to the identification of the first plant gene underlying a tomato QTL for fruit size (Grandillo et al., 1999; Frary et al., 2000).

With the increasing number of linkage mapping studies of *R* genes and quantitative resistance loci (QRL) using tomato and potato RFLP and microsatellite markers of known chromosomal position, it became possible to combine the positional information generated in independent mapping experiments into a single function map for pathogen resistance (Grube et al., 2000; Gebhardt and Valkonen, 2001; Danan et al., 2011). However, positions and distances between loci based on recombination frequencies have lower precision compared with physical positions and distances measured in DNA base pairs. This is due to the large variability of linkage map resolution with the type and size of mapping populations, marker density, and suppressed recombination in centromeric regions. When sequence information is available for DNA-based markers used in linkage mapping experiments, it is now possible to use whole genome reference sequences available in databases (http://spuddb.uga.edu/, https://solgenomics.net/) to construct physical chromosome maps, which combine the positional information of phenotypic characters from independent linkage mapping experiments. In addition, several hundred candidate genes for the traits were available in the literature that could be included in the physical maps. Such maps should facilitate comparisons of the genomic positions of phenotypic characters across species borders, eventually pointing to a common molecular basis of traits mapping to syntenic positions and genomic regions of particular interest.

This paper provides a survey and database of more than four hundred publications on linkage and association mapping of qualitative and quantitative traits with DNA-based markers, and map-based cloning of genes underlying such traits, primarily in potato and tomato and to a lesser extent in pepper and eggplant. The information provided in the literature was used to construct the first physical map of qualitative and quantitative traits on the 12 chromosomes of tomato and potato, including some resistance traits of pepper and eggplant. The purpose of this paper is to summarize 40 years of research in this specific field and to provoke new thoughts and hypotheses about the organization and identity of genes controlling important agronomic characters in crop plants.

## 2 Methods

Sequences of potato RFLP markers derived from randomly selected genomic (GP***) and cDNA clones (CP***, S****, and P****) (Gebhardt et al., 1989; 1994) were retrieved from the GenBank NCBI (accession numbers in Supplementary Tables S1–S12). Tomato RFLP markers were randomly selected genomic (TG***) or cDNA clones (CT*** or CD***) (Tanksley et al., 1992). Most tomato RFLP marker sequences were available in the Sol Genomics Network (SGN) database (https://solgenomics.net/search/markers). Tomato marker sequences not available in SGN were eventually retrieved from NCBI (Ganal et al., 1998). Primer sequences for PCR-based markers were taken from the literature or the SGN database (tomato microsatellites). AFLP and RAPD markers were not used due to insufficient sequence information. Sequences flanking tomato SNPs (solcap_snp_sl_*****) were obtained from the SGN database. Sequences flanking potato SNPs (solcap_snp_c*_*****) were obtained from http://solcap.msu.edu/potato and the SPUD database (http://spuddb.uga.edu/). Sequences flanking other SNPs were taken from the corresponding reference paper. Gene sequences were retrieved via accession number from NCBI, SPUD, and SGN databases and in some cases directly from the corresponding article. Gene sequences of *Arabidopsis thaliana* were retrieved from the Arabidopsis information resource (TAIR) database (https://www.arabidopsis.org/). The source of each sequence is provided in Supplementary Tables S1–S12.

DNA sequences or translated polypeptide sequences, for example, *A. thaliana* sequences, were mapped *in silico* to the genome sequences of potato and tomato (potato genome versions DM v4.03 and DM_v6.1; tomato genome version SL4.0) using the BLAST tool in the SPUD and SGN databases and default parameters. For short sequences, e.g., PCR primers, the parameter “expect threshold” was increased to 1. The physical position of markers and genes was determined in most cases unambiguously based on high sequence similarity (>70% identity). In cases of multiple sequence matches, the chromosomal position of a marker or gene known from genetic mapping was used to identify the most likely physical position.

Qualitative and quantitative traits were mapped based on the information content of articles published from 1988 to 2022. Articles were selected using the following criteria: articles that established the first molecular linkage maps of tomato and potato with RFLP and microsatellite markers, which were subsequently used for trait mapping; articles on linkage and association mapping of pathogen resistance and QTL for sugar content, yield, and maturity; articles on the cloning of resistance genes and QTL in the Solanaceae. Excluded were articles on mapping exclusively with AFLP and RAPD markers, redundant articles not adding further positional information to a specific locus, a few articles with very unclearly presented results, and articles in which the reported map position of the trait contradicted the physical map position. Traits with known molecular basis (cloned resistance genes and QTL) were physically mapped using the NCBI accessions. The position and size of genomic segments harbouring the genes controlling traits with unknown molecular basis were estimated based on the physical positions of linked, associated, and, whenever possible, flanking markers. The information from multiple mapping experiments performed with different mapping populations and different markers was combined. The physical borders of QTL could not always be precisely determined based on flanking markers and are approximations. For example, when only one linked marker from one experiment was available, it was assumed that the linked QTL was located within 2 Mbp upstream and downstream of the marker. This might have led to an overestimation of the size of a physical genome segment toward chromosome ends and an underestimation in centromeric regions. QTL resulting from epistatic interactions were not considered.

## 3 Results and discussion

The plant material used for the linkage mapping of phenotypic characters in the self-compatible, inbred tomato was segregating F2 and backcross populations, near-isogenic lines (NILs) and recombinant inbred lines (RILs). Progeny was derived from the hybridization of cultivars with wild tomato species (Supplementary Table S13). In pepper, inbred cultivars and a few wild species (*C. chinense, C. frutescens, C. baccatum, and C. chacoense*) were hybridized to generate inter- and intra-specific F_2_ or doubled haploid (DH) populations. The cultivated potato is tetraploid with tetrasomic inheritance, which complicates the linkage analysis. Varieties and cultivars are heterozygous due to breeding schemes based on outcrossing. Linkage mapping in potato was mostly performed in F_1_ progeny of diploid, self-incompatible, and, therefore, heterozygous parents. Most of them contained in their pedigree one or more introgressions from wild tuber-bearing *Solanum* species (Supplementary Table S13). The genetics of this material is equivalent to the genetics of human families and has the advantage that up to four alleles at each locus segregate in the progeny. The discovery of the *Sli* (S-locus inhibitor) gene, which confers self-compatibility to diploid potato plants (Hosaka and Hanneman, 1998a; Hosaka and Hanneman, 1998b) marks the beginning of a new approach in potato genetics and breeding based on inbred lines, similar to tomato (Lindhout et al., 2011; Kaiser et al., 2020). However, such material has no significant role yet in the literature on the genetic mapping of agronomic traits. At the beginning of the 21st century, association or linkage disequilibrium mapping (Zhu et al., 2008; Burghardt et al., 2017) of quantitative trait loci (QTL) in populations of cultivars started to supplement linkage mapping based on experimental populations (Gebhardt et al., 2004; Simko et al., 2004; Mazzucato et al., 2008).

Based on publicly available DNA sequence information, I constructed physical maps of the 12 potato and tomato chromosomes, first with genetically mapped RFLP and microsatellite markers (Bonierbale et al., 1988; Gebhardt et al., 1991; Tanksley et al., 1992; Milbourne et al., 1998; Gebhardt et al., 2003; Feingold et al., 2005; Ghislain et al., 2009) and second with DNA-based markers of various types (RFLP, SSR, SCAR (Sequence Characterized Amplified Region), CAPS (Cleaved Amplified Polymorphic Sequence), KASP (Kompetitive Allele Specific PCR), and SNP (Single Nucleotide Polymorphism)) that were linked or associated with genetic factors for pathogen resistance, the yield and the sugar content of tomato fruits and potato tubers, and maturity. Furthermore, candidate genes, cloned genes for pathogen resistance, and some other cloned genes encoding QTL, self-incompatibility (potato), and colour traits (Supplementary Tables S1–S12) were included in the maps

### 3.1 Comparison of potato and tomato genome structure

Matching pairs of physical positions both in the potato and tomato genome were found for 2,741 sequence-based loci (Supplementary Tables S1–S12). In the case of physical clusters of highly similar sequences, for example, NBS-LRR (nucleotide-binding site–leucine-rich repeat)-type gene families, the matching pairs within the clustered gene family were sometimes ambiguous. This was without consequences for the higher-order structural comparisons. No matching pair of physical positions in both potato and tomato were found for 121 genetically mapped loci. The reasons were insufficient or lack of sequence similarity, high sequence redundancy, or possible sequencing errors or errors in genetic mapping or genome assembly. For similar reasons, no matching pair of physical positions either in potato or tomato was found for 321 loci (Supplementary Tables S1–S12). Some of these cases might indicate small genomic rearrangements between potato and tomato, which were not reliably resolved with the density of physical loci, especially in centromeric regions. Inconsistencies between genetic and physical marker order were also observed (Supplementary Tables S1–S12), most likely due to imprecision and errors in genetic mapping or, alternatively, due to genome assembly errors.

Physical locus density was much higher on the chromosome arms comprising approximately 50% of the whole genome as compared with central regions (Figures 1–12; Supplementary Tables S1–S12). This was similar to the density distribution of annotated genes in the genome sequences (genome browsers in SGN and Spud DB). Consequently, the majority of genetically mapped traits were also located on the chromosome arms.

**FIGURE 1:**
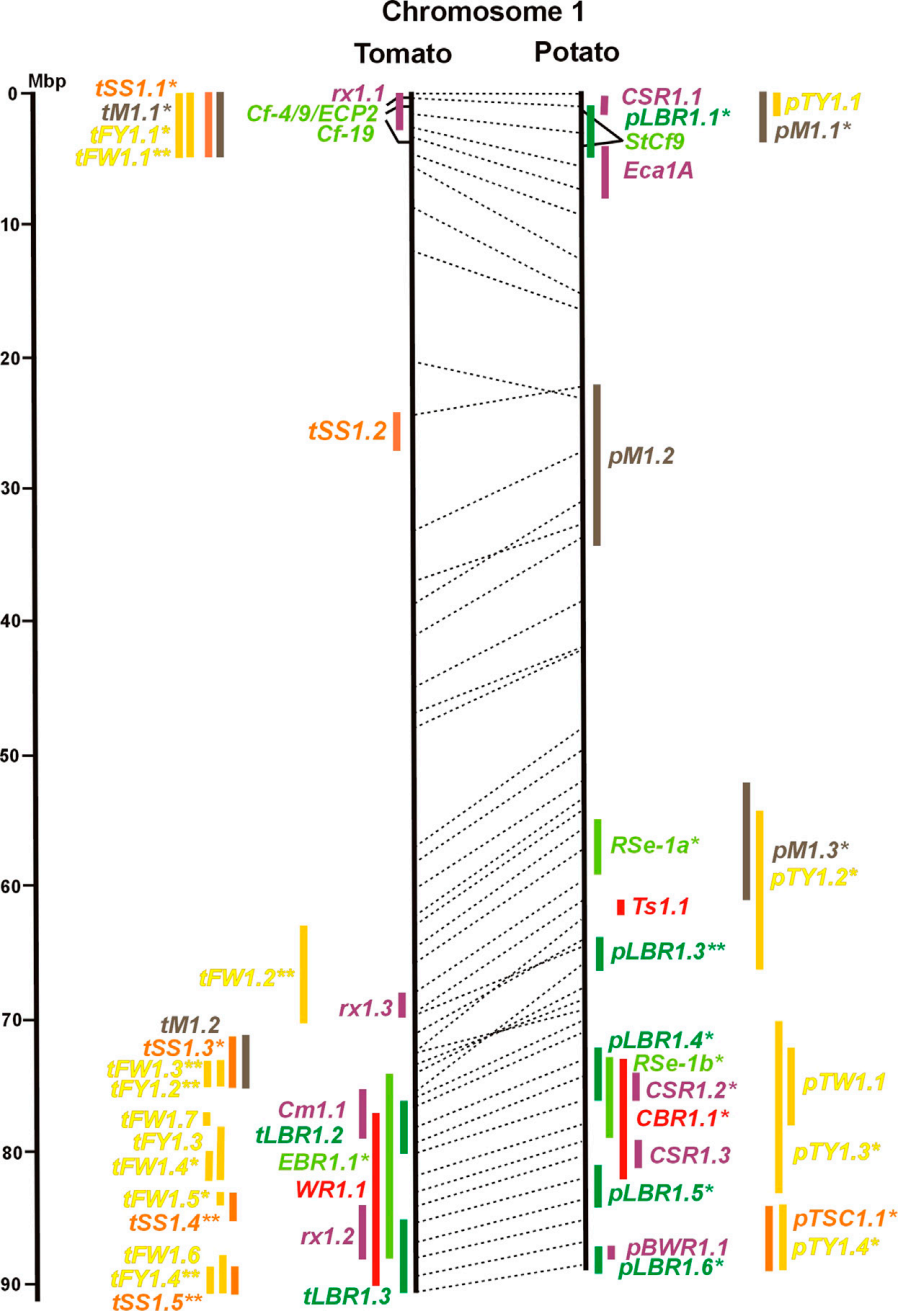
Physical maps in megabase pair scale (Mbp) of the 12 tomato/potato chromosomes. Genome version SL 4.0 and DM v6.1 of tomato and potato, respectively, are the basis of the physical chromosome maps represented by vertical black lines. The genome sequences of the chromosomes have the same orientation, except chromosome 12, where the tomato sequence is inversely oriented *versus* the potato sequence. Each tomato chromosome map is linked to the corresponding potato chromosome map by dotted lines representing the positions of a subset of sequence-based markers (indicated by * in column A of Tables S1 to S12). Clear intrachromosomal translocations are indicated by red dotted lines. The positions of sequenced genes for pathogen resistance and QTL are shown by a single black line connecting the chromosome map with the gene’s colour-coded name to the left (tomato) or right (potato) of the map. Intervals including mapped but not sequence-characterized qualitative resistance genes are shown as two black lines representing the borders that connect the chromosome map with the gene’s colour-coded name. Positions of pepper and eggplant resistance genes are shown at syntenic positions on both maps. QRL for pathogen resistance and QTL for sugar content, yield, and maturity are shown as colour-coded bars to the left (tomato) or right (potato) of the chromosome map. QRL and QTL names (for nomenclature see text and Tables 1 and 3) are shown in the same colour next to the coloured bar. Colour codes: Dark green–resistance to the oomycete *P. infestans*; light green–resistance to fungi; purple–resistance to bacteria; blue–virus resistance; magenta–nematode resistance; red–insect resistance; orange–QTL for fruit and tuber sugar content; yellow–QTL for fruit and tuber weight and yield; maroon–QTL for maturity. QRL and QTL names extended with * were supported by two or three mapping studies; QRL and QTL names ending with ** were supported by more than three mapping studies. QRL and QTL names without an extension were supported by a single mapping study. Coordinates in base pairs for all loci are provided in Tables S1 to S12.

**FIGURE 2:**
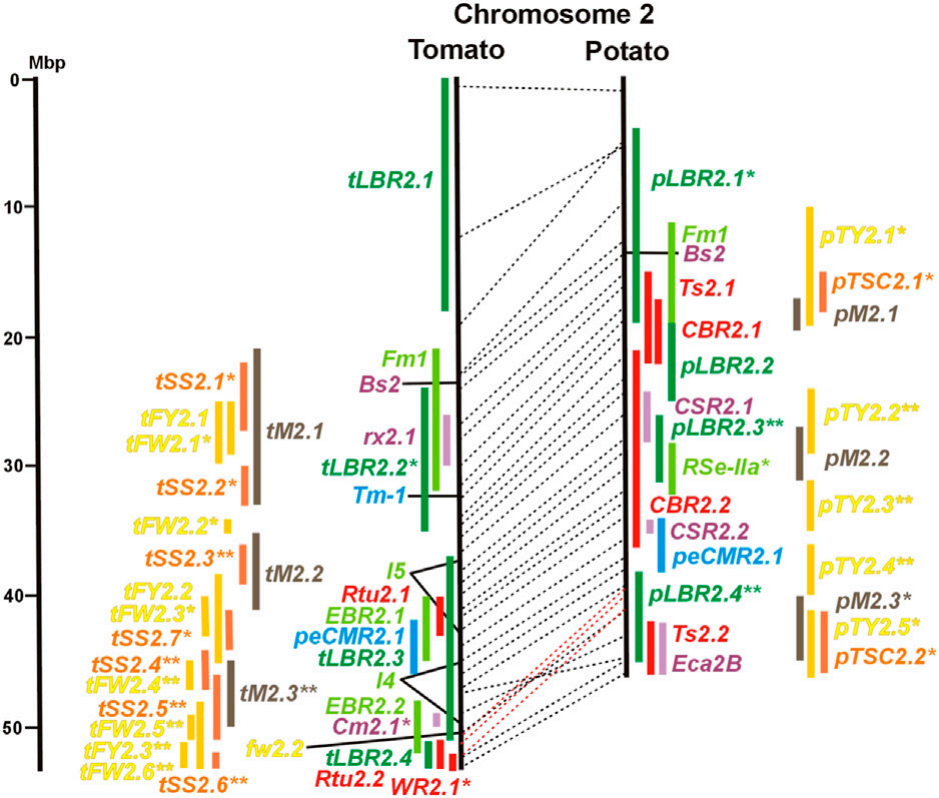
See caption for Figure 1 for detailed description.

**FIGURE 3:**
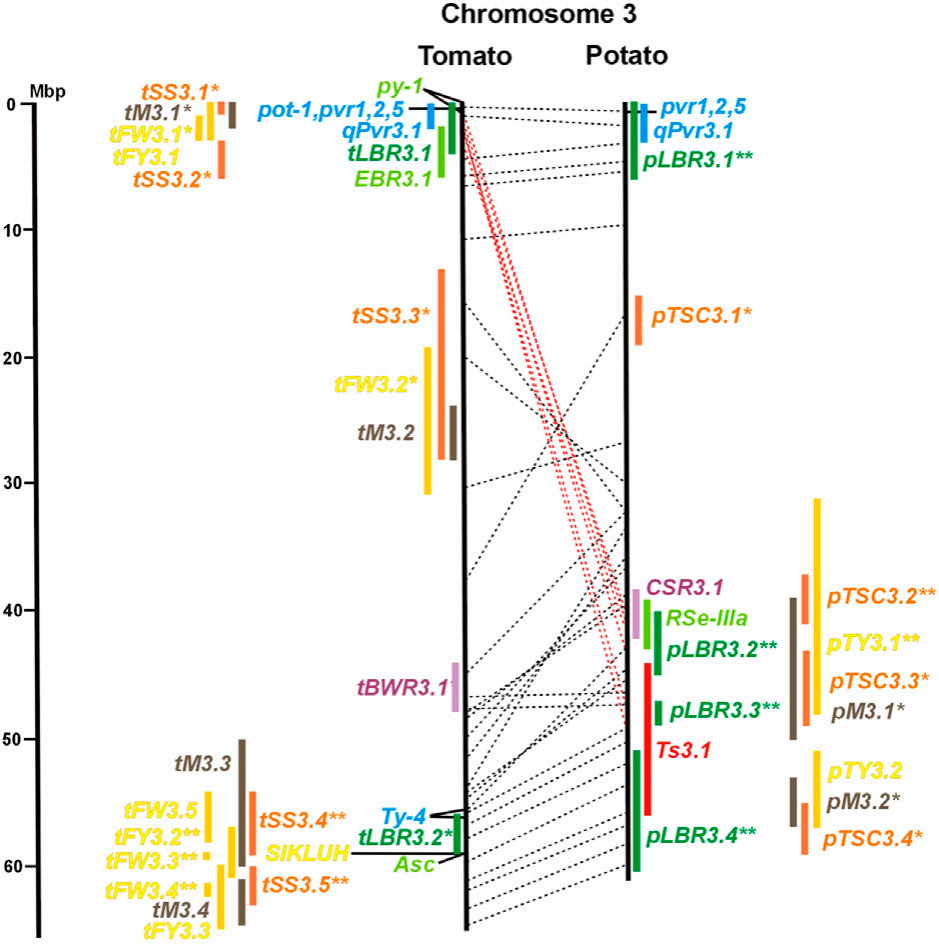
See caption for Figure 1 for detailed description.

**FIGURE 4:**
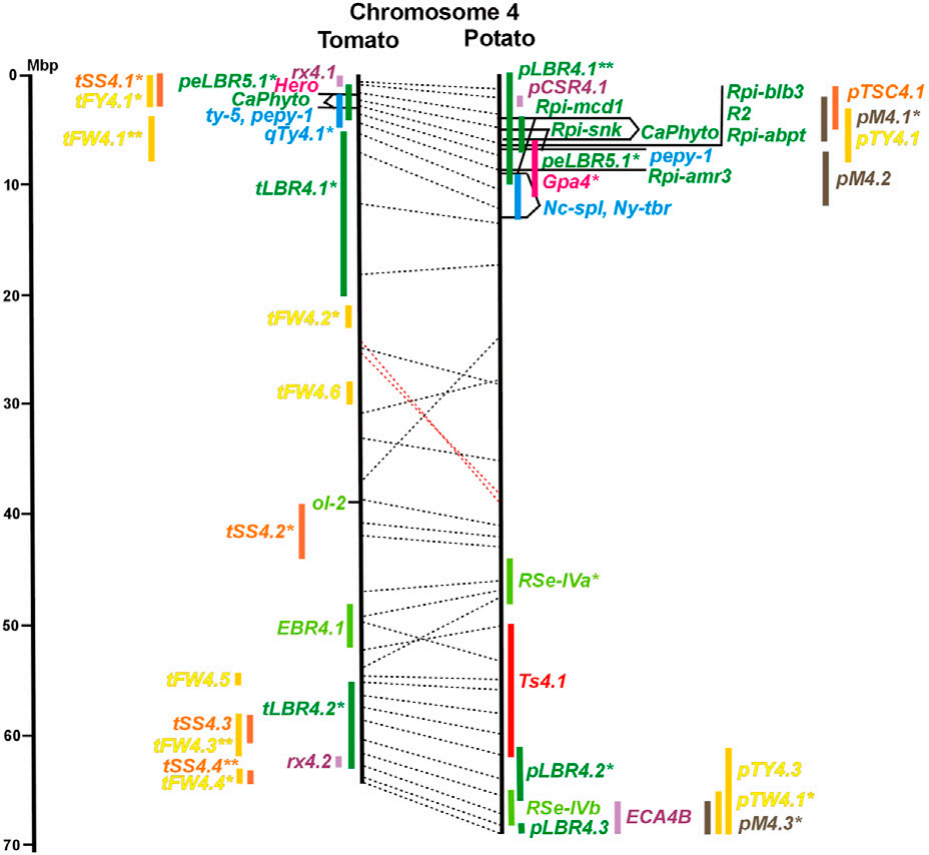
See caption for Figure 1 for detailed description.

**FIGURE 5:**
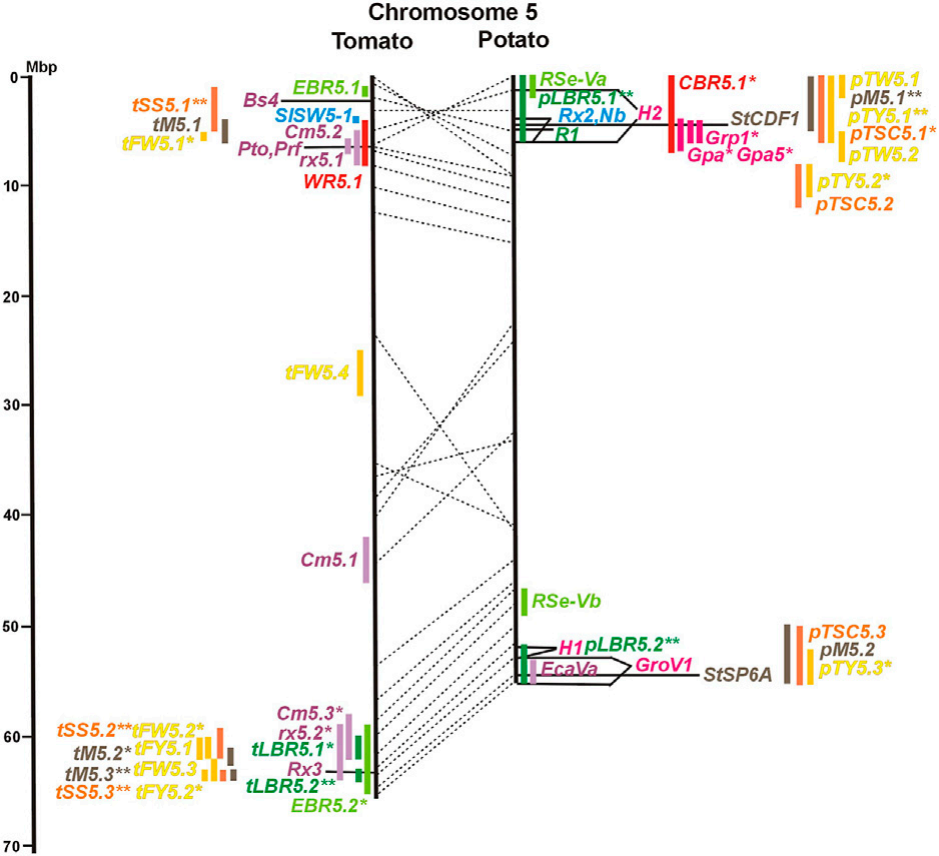
See caption for Figure 1 for detailed description.

**FIGURE 6:**
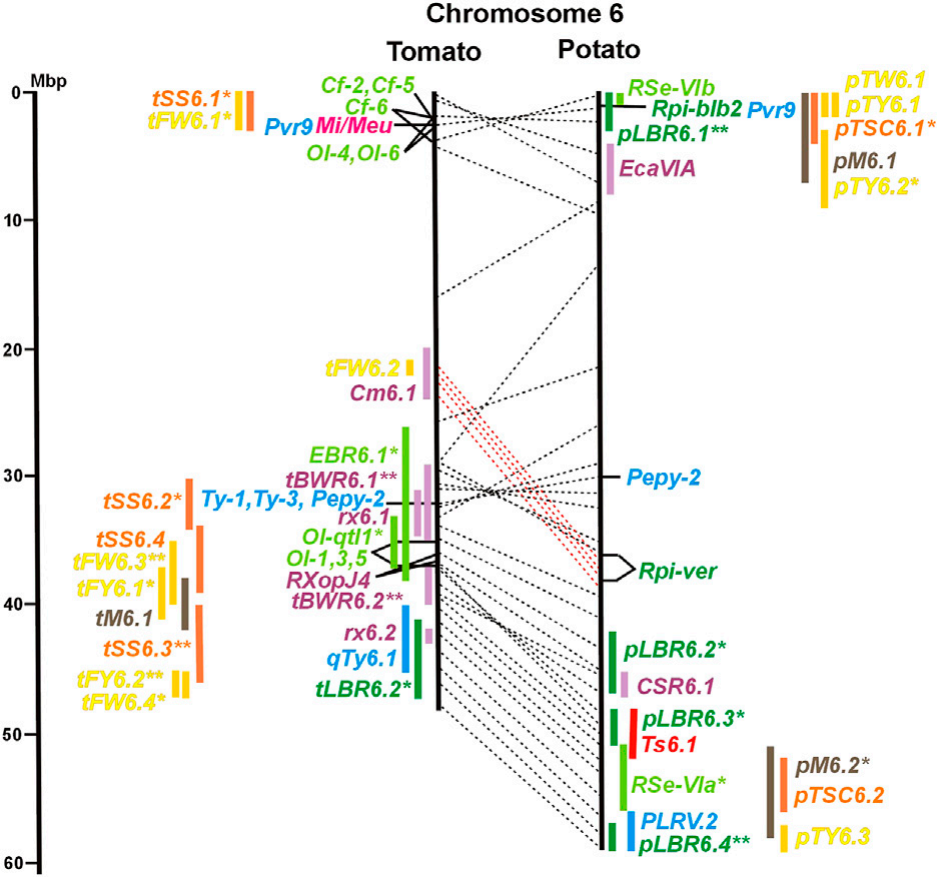
See caption for Figure 1 for detailed description.

**FIGURE 7:**
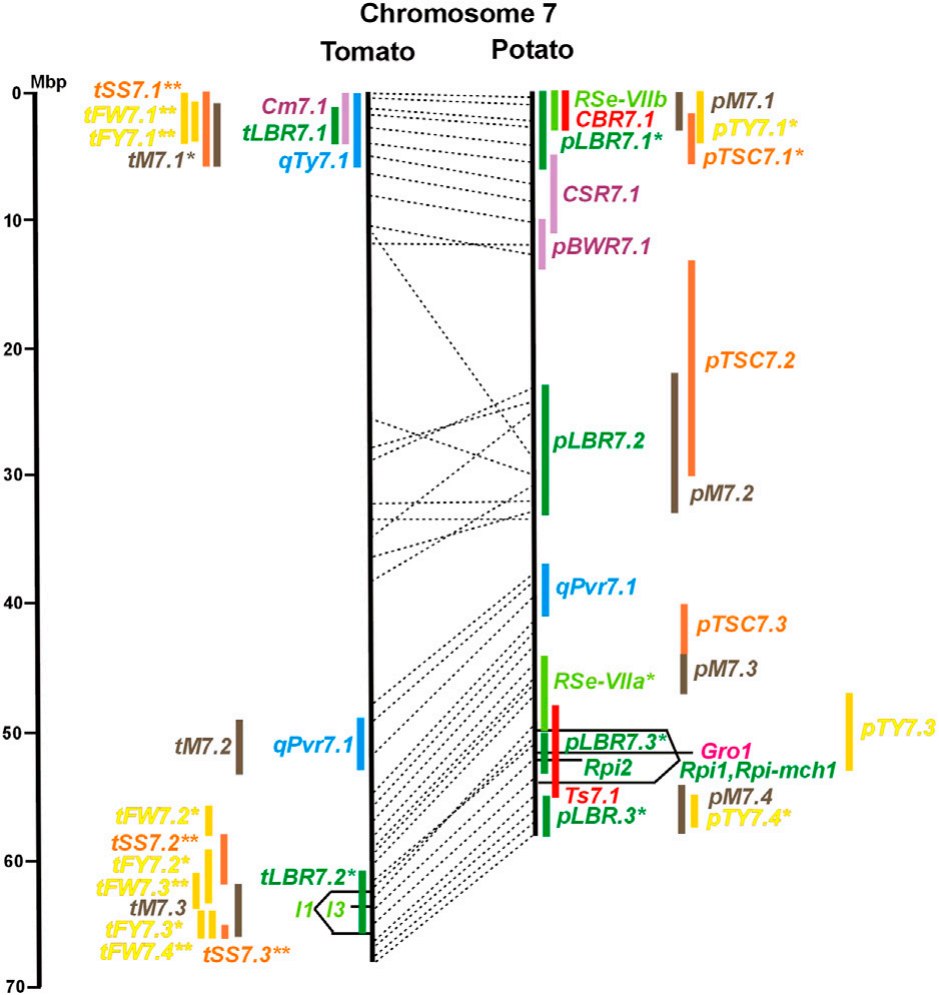
See caption for Figure 1 for detailed description.

**FIGURE 8:**
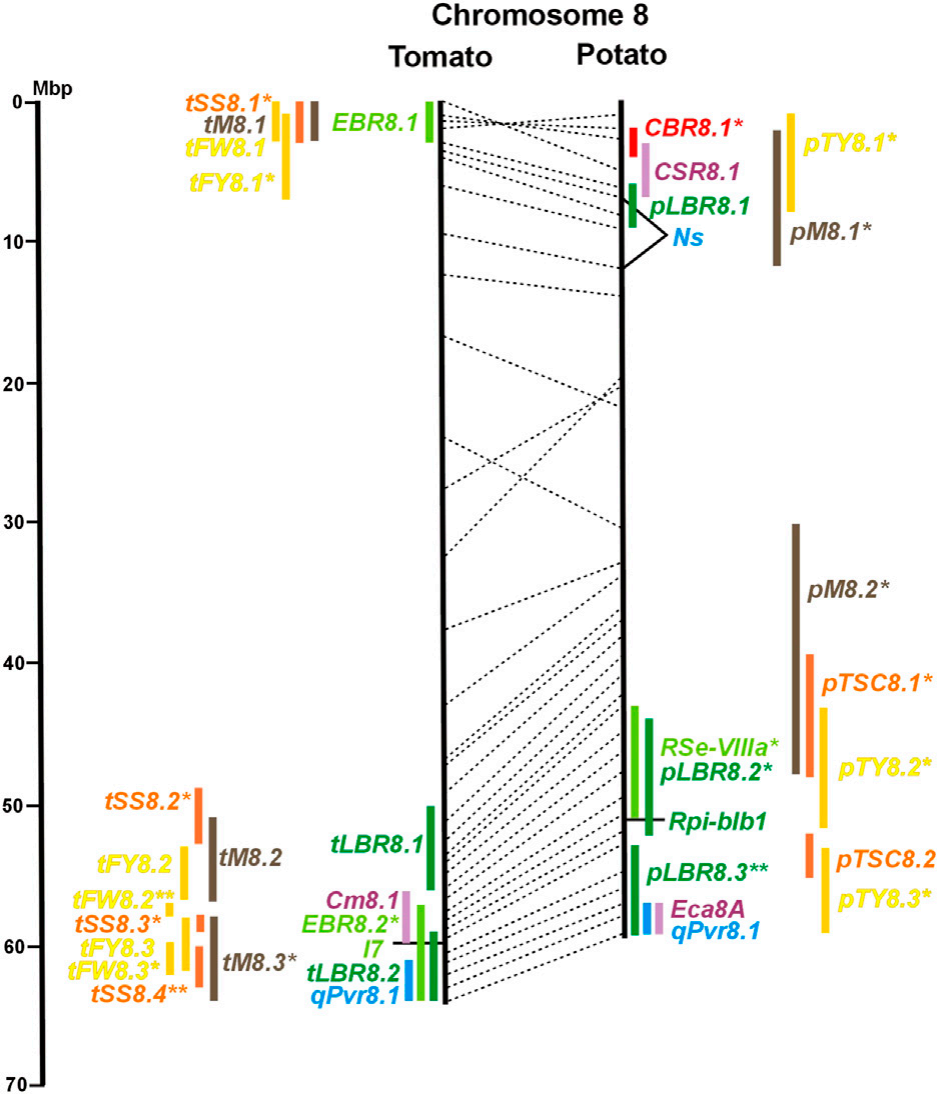
See caption for Figure 1 for detailed description.

**FIGURE 9:**
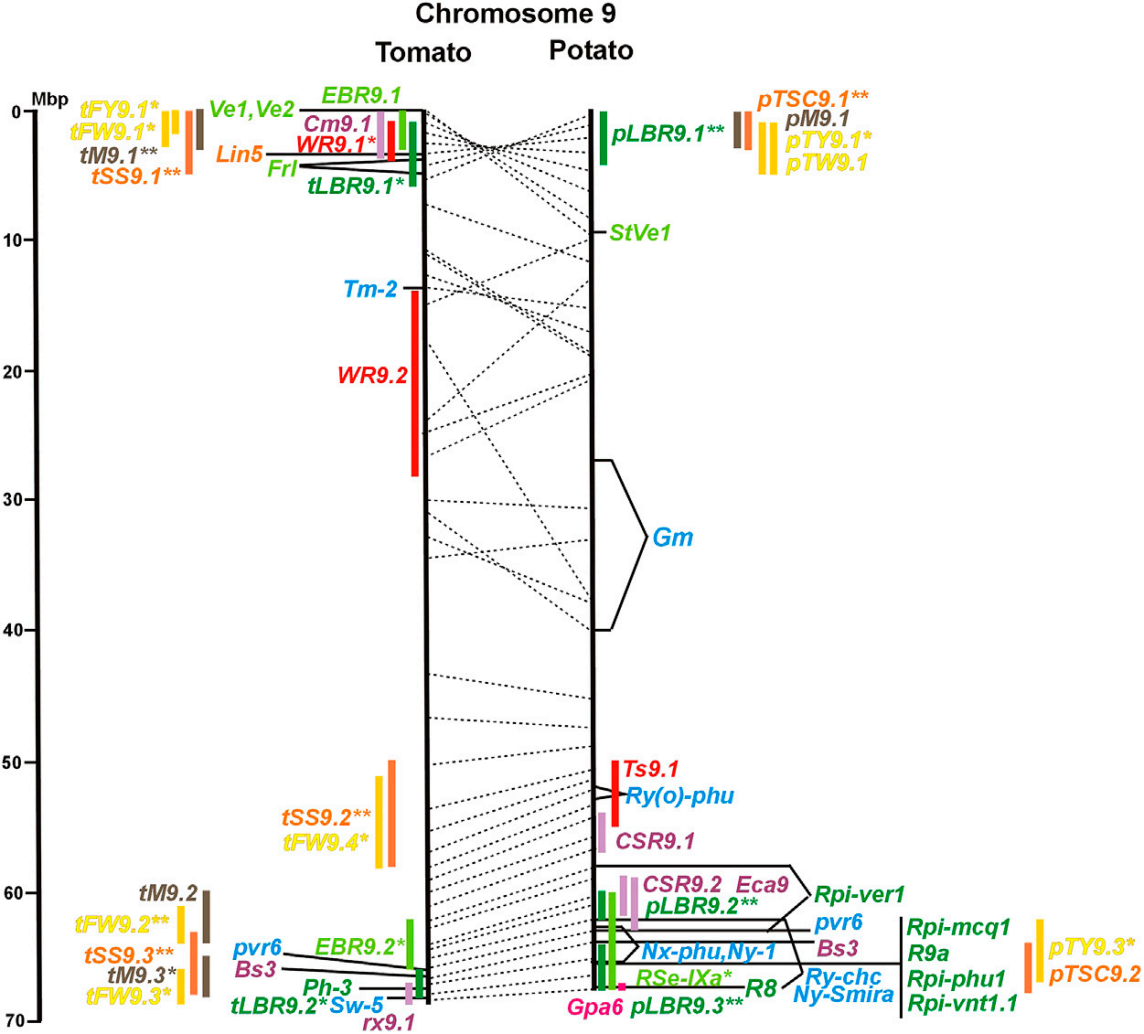
See caption for Figure 1 for detailed description.

**FIGURE 10:**
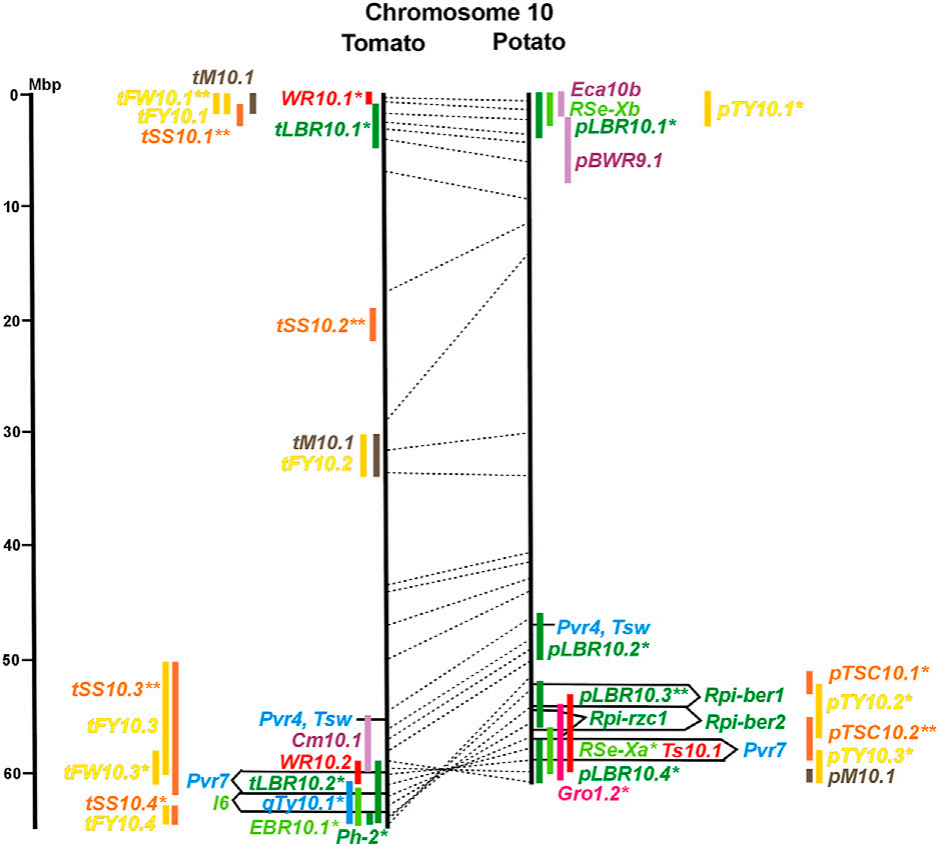
See caption for Figure 1 for detailed description.

**FIGURE 11:**
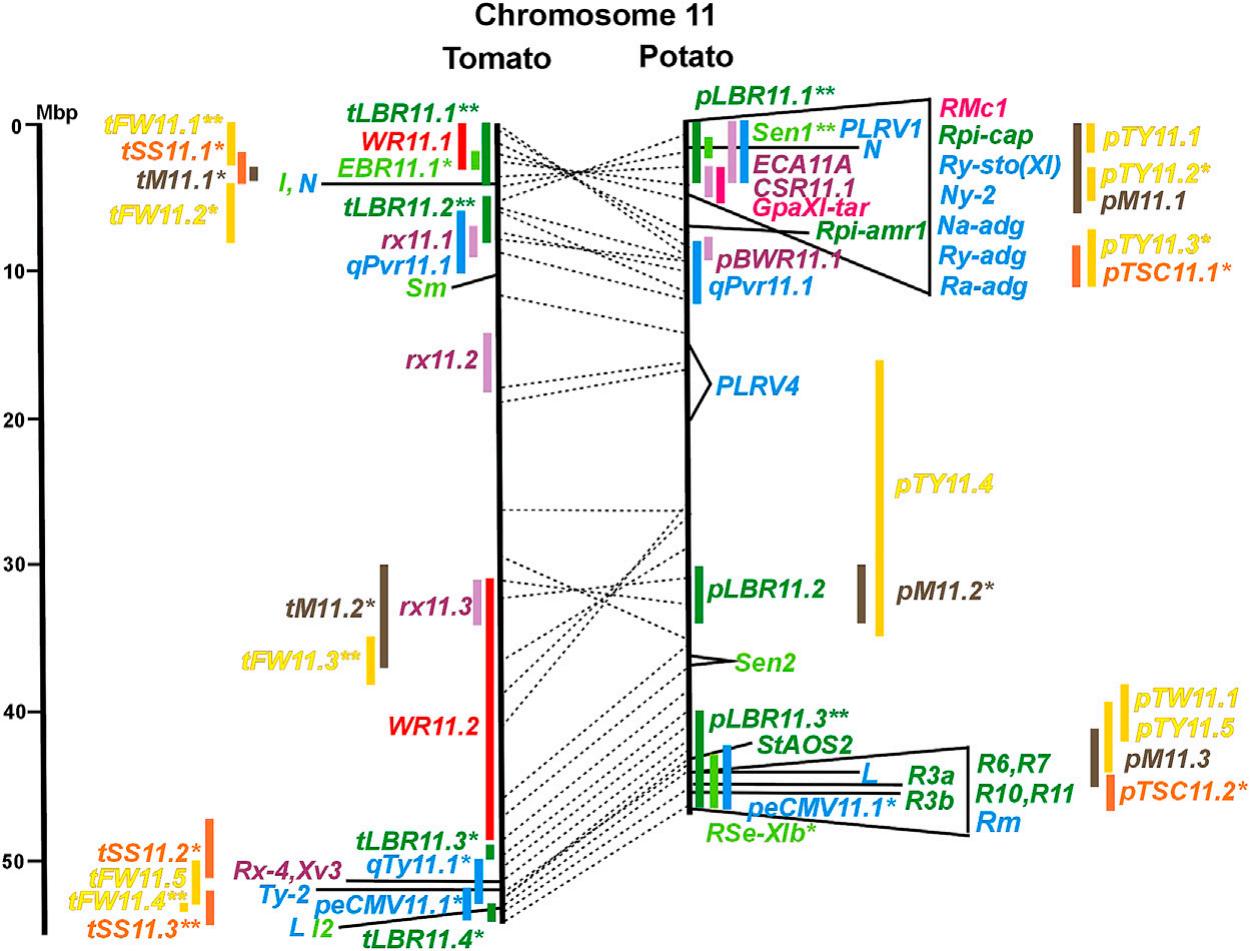
See caption for Figure 1 for detailed description.

**FIGURE 12:**
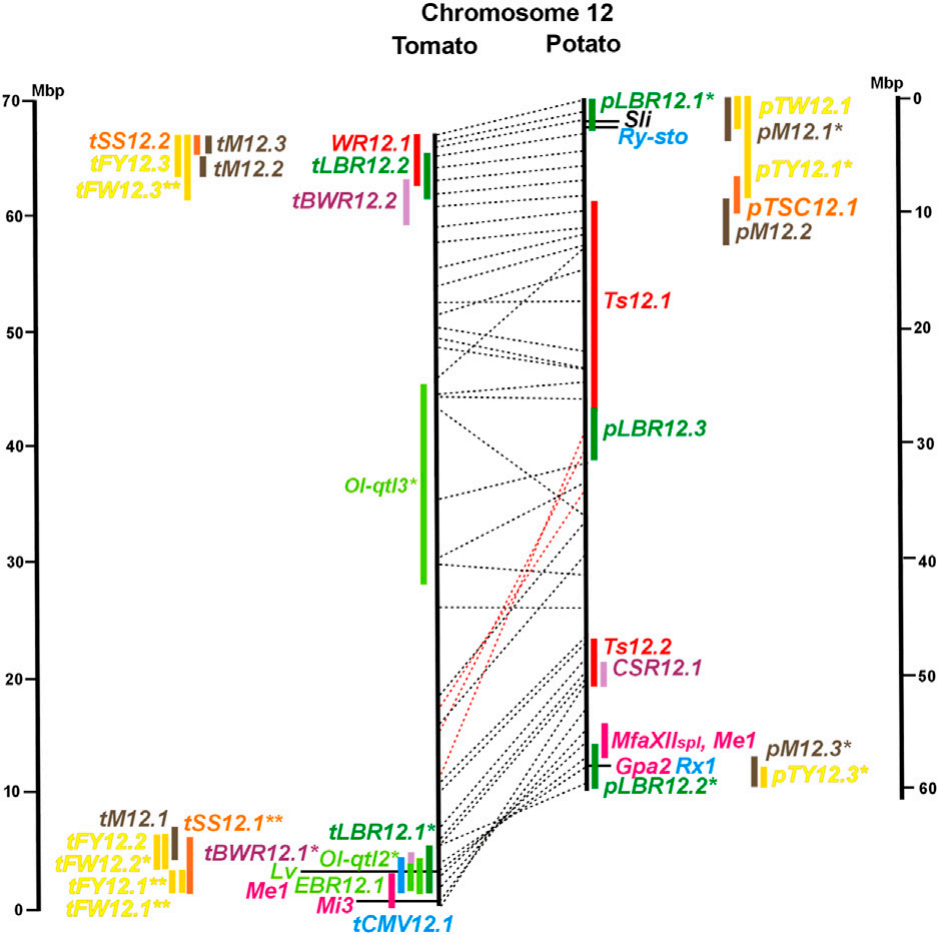
See caption for Figure 1 for detailed description.

Comparative molecular linkage maps of tomato and potato have been constructed (Bonierbale et al., 1988; Tanksley et al., 1992; The Tomato Genome Consortium, 2012). They have low resolution compared with physical maps. Comparison based on genomic sequences has the highest resolution. However, the single comparison of this type (The Tomato Genome Consortium, 2012) used primary versions of both the tomato and potato genome sequences, where uncertainty about the orientation of physical contigs still existed. The comparison presented in this paper is based on the physical mapping of 2,741 short sequences to consolidated reference genome sequences of tomato and potato (tomato version SL4.0; potato version DM v6.1). It has an intermediate resolution. Besides the eight genetically or cytogenetically resolved intra-chromosomal inversions between tomato and potato chromosomes 2, 5, 6, 9, 10, 11, and 12 (Tanksley et al., 1992; The Tomato Genome Consortium, 2012), 25 additional inversions and 12 intra-chromosomal translocations with a size range from 0,2–22 megabase pairs (Mbp) were detected (Figures 1–12; Supplementary Tables S1–S12). The translocation and inversion on chromosome 2, the inversion on the short arm of chromosome 6, and the inversion on the long arm of chromosome 10 are consistent with cytogenetic FISH analyses (Peters et al., 2012). Twelve inversions and six translocations were located in central chromosomal regions with low genetic and physical resolution. Some of those might be artefacts of the genome assembly in either tomato or potato. Chromosomes 3, 6, and 11 carried the most rearrangements. Traces of ancient intrachromosomal duplications predating the speciation of tomato and potato were detected on chromosomes 2, 6, and 8 based on the duplication of linked loci in both species, which mapped to two different physical sections of the same chromosome (Gebhardt et al., 2003).

### 3.2 Physical chromosome maps of qualitative and quantitative pathogen resistance

Three hundred and six articles were analysed, which describe the molecular mapping, map-based cloning, and characterization of loci for qualitative (monogenic) and quantitative (polygenic) resistance (quantitative resistance locus QRL) to plant pathogenic oomycetes, fungi, viruses, bacteria, nematodes, and insects in potato (150 articles), tomato (130), pepper (23), eggplant (2), and tobacco (1) (Supplementary Data Sheet S14 and Supplementary Table S15). The phenotypic distinction between qualitative and quantitative resistance was not always clear-cut. The analysis of an observed phenotypic distribution with quantitative statistics may result in one QRL with a major effect, indicating that the resistance is based on a single locus. Vice versa, the distinction between resistant and susceptible phenotypic classes might be blurred by variation within the classes. The literature exhibited large diversity with respect to size and type of mapping populations and genome coverage with DNA-based markers from less than a hundred RFLPs to thousands of SNPs, environments, and methods to assess resistance phenotypes. Nevertheless, in a number of cases, QRL genetically mapped in independent studies were allocated to the same genome segment, suggesting that they eventually tag the same gene(s). The nomenclature used in different studies for QRL to the same pathogen was also diverse and inconsistent. To integrate the information from various sources and make it more accessible, a uniform nomenclature was adopted for some quantitative resistance traits (Table 1). The same name was assigned to a QRL when it was allocated to a similar genome segment in different studies. The originally published locus names, as far as they were assigned, are included in Supplementary Tables S1–S12.

**TABLE 1:** Number of physically mapped QTL for pathogen resistance in potato, tomato, pepper, and eggplant.

Pathogen class	Pathogen	QRL acronym	Potato	Tomato	Pepper	Eggplant
Oomycetes	*Phytophthora infestans, P. capsici* (late blight)	*LBR*	43	29	1	
Fungi	*Alternaria solani* (early blight)	*EBR*		15		
	*Oidium lycopersicum* (powdery mildew)	*Ol-qtl*		3		
	*Synchytrium endobioticum* (potato wart)	*RSe*	19			
	*Verticillium dahliae* (Verticillium wilt)	*Ve*	1	1		
	*Fusarium oxysporum* (Fusarium wilt)	*Fm1*				1
Bacteria	*Clavibacter michiganensis* (bacterial canker)	*Cm*		10		
	*Erwinia (Pectobacterium) carotovora subsp. atroseptica* (blackleg and soft rot)	*Eca*	9			
	*Ralstonia solanacearum* (bacterial wilt)	*BWR*	5	8		2
	*Streptomyces scabies* (common scab)	*CSR*	15			
	*Xanthomonas spp* (bacterial spot)	*rx*		14		
Viruses	Begomovirus, *Tomato Yellow Leaf Curl Virus* (TYLCV)	*qTy*		6		
	*Cucumber Mosaic Virus* (CMV)	*CMV*		1	2	
	*Potato Leafroll Virus* (PLRV)	*PLRV*	2			
	Potyvirus, *Potato Virus Y* (PVY)	*qPvr*			4	
	Tospovirus, *Tomato Spotted Wilt Virus* (TSWV)	*SlSW5-1*		1		
Nematodes	*Globodera pallida*	*Gpa*	6			
	*Globodera rostochiensis*	*Gro*	2			
Insects	*Bemisia tabaci,* (whitefly)	*WR*		10		
	*Leptiotarsa decemlineata* (Colorado beetle)	*CBR*	8			
	*Tecia solanivora* (potato tuber moth)	*Ts*	12			
	*Tetranychus urticae* (two-spotted spider mite)	*Rtu*	2			

Taxonomic names of species are written in italic.

The frequency of genetic studies dealing with plant resistance to a specific pathogen corresponds to some extent with the importance of this pathogen for crop cultivation. On the other hand, studies on important resistance traits might be underrepresented due to the difficulties in analysing the resistance phenotype, for example, insect resistance and resistance to potato wart. The most studied pathogen resistance in potato was resistance to the oomycete *Phytophthora infestans*, followed by resistance to the root cyst nematodes *G. rostochiensis* and *Globodera pallida,* and viruses, particularly, *Potato Virus Y* (PVY) (Supplementary Data Sheet S14). In tomato, resistance to various fungi and bacteria was important besides resistance to *P. infestans*, followed by virus resistance, particularly, to *Tomato Yellow Leaf Curl Virus* (TYLCV), and resistance to the nematode *M. incognita*. Despite the close phylogenetic relationship between potato and tomato, only six of 33 host-pathogen systems were analysed in both species (*P. infestans*, *Verticillium dahliae, Ralstonia solanacearum*, Potyvirus, *Globodera rostochiensis, and Meloidogyne incognita*) (Supplementary Data Sheet S14). Resistance to *Phytophthora* species, *Potato Virus Y* (PVY), and root-knot nematodes (*Meloidogyne* sp.) was analysed in potato, tomato, and pepper. Resistance to *R. solanacearum* was analysed in tomato, potato, and eggplant, and resistance to *Tobacco Mosaic Virus* (TMV) in tomato, pepper, and tobacco. Resistance to *Xanthomonas* species and the viruses *Tomato Yellow Leaf Curl Virus* (TYLCV) and *Cucumber Mosaic Virus* (CMV) was analysed in both tomato and pepper (Supplementary Data Sheet S14). This shows the relevance of these host-pathogen systems across species borders in the Solanaceae family. Compared with potato and tomato, fewer genetic studies on pathogen resistance have been published in pepper and eggplant. Only some of these results could be integrated into the physical maps of potato and tomato due to the more fragmented synteny of pepper and eggplant compared with tomato and potato. Mainly, single pepper genes for virus resistance and some major QRL could be placed on the physical potato/tomato maps. The sequence of the tobacco *N* gene for resistance to the *Tobacco Mosaic Virus* (TMV) was also available. *N* is a prototype of the major class of plant genes for pathogen resistance characterized by a nucleotide-binding site (NBS) and a leucine-rich repeat (LRR) domain (Whitham et al., 1994).

### 3.3 Monogenic pathogen resistance

DNA sequences of 57 resistance genes were available in the literature, mostly but not exclusively dominant *R* genes. They were introgressed into cultivars from wild *Solanum* species. Twenty-seven resistance genes were from tomato, twenty from potato, nine from pepper, and one from tobacco. With two exceptions, all resistance genes were placed on syntenic physical positions in the tomato and potato genome (Figures 1–12; Supplementary Tables S1–S12). No sequence homology was found on chromosome 5 of potato and tomato for the potato *Rx2* gene for resistance to *Potato Virus X* (PVX). *Rx2* originated from *S. acaule*, and it was mapped on potato chromosome 5 (Ritter et al., 1991) and cloned (Bendahmane et al., 2000) (Figure 5; Supplementary Table S5). It seems unlikely that both the potato and tomato genome assemblies should have sequence gaps at the same position. Therefore, orthologous genes of *Rx2* on chromosome 5 are obviously absent in the genotypes of *S. phureja* and *S. lycopersicum,* from which the reference genomes were constructed. Furthermore, the potato homologs of the tomato resistance gene *Sm* on chromosome 11 mapped to non-syntenic positions on the same chromosome (Supplementary Table S11).

Two-thirds of the sequence-characterized resistance genes belong to the major class of NBS-LRR type plant genes for pathogen recognition (reviewed in Andersen et al., 2018). The remaining third includes six recessive resistance genes, three of tomato (*pot-1, ty-5, ol-2*) and three of pepper (*pvr1,2,5, pepy-1, and pvr6*). The ‘atypical’ resistance genes represent diverse structures and functions, among others a protein kinase (*Pto* on chromosome 5) (Martin et al., 1993a; Martin et al., 1993b), the eukaryotic translation initiation factor 4E (*pvr2 and pot-1* on chromosome 3) (Ruffel et al., 2002; Ruffel et al., 2005), and RNA-dependent RNA polymerase (*Ty-1, Ty-3, and Pepy-2* on chromosome 6) (Verlaan et al., 2013; Koeda et al., 2022).

Fifty-six mapped resistance genes with yet unknown sequences were located on the physical potato/tomato maps by means of linked and flanking markers, thirty-six of potato, seventeen of tomato, and three of pepper (Figures 1–12; Supplementary Tables S1–S12). Fifty-five genes mapped to chromosome arms in physical intervals between 40 Kbp and 6 Mbp with an average of 2,5 Mbp. Only one resistance gene (*Gm* on potato chromosome 9) mapped to a physical interval >10 Mbp in the centromeric region.

Many qualitative resistance loci colocalized with quantitative resistance loci for the same or other pathogens (see below).

### 3.4 Polygenic and monogenic resistance to pathogenic oomycetes, fungi, bacteria, viruses, nematodes, and insects

Based on the sequences of linked, associated, and flanking DNA markers, the position of 230 quantitative resistance loci (QRL) (120 in potato, 100 in tomato, 7 in pepper, and 3 in eggplant) on the 12 physical tomato/potato chromosome maps was estimated (Table 1). Sixteen QRL (nine in tomato and seven in potato) were mapped to physical intervals larger than 15 Mbp and were not further considered in comparisons within and between the species. The remaining 214 QRL mapped to intervals between 0,5 and 15 Mbp with an average of 4,2 Mbp in both tomato and potato. Physical intervals of this size contain hundreds of genes. One or more genes may be responsible for any mapped QRL. The number of genes underlying quantitative resistance is therefore certainly higher than the actual number of mapped QRL.

#### 3.4.1 Resistance to the oomycete *Phytophthora infestans*

The oomycete *P. infestans* causes late blight disease in potato and tomato, which can lead to complete crop loss. To reduce the necessity of chemical control, late blight-resistant cultivars are important breeding goals. Qualitative and quantitative resistance to late blight was most extensively studied in potato (Supplementary Data Sheet S14). Twenty-eight *R* genes were mapped on eight chromosomes, fifteen of which were cloned and sequenced (Figures 1–12; Supplementary Tables S1–S12, and Supplementary Table S15). Besides *R* genes, quantitative or field resistance to late blight was extensively analysed in potato (33 articles in potato, 12 in tomato, and 2 in pepper (Supplementary Data Sheet S14). Resistance levels were quantified based on different *P. infestans* races and various evaluation methods, such as detached leaf assays (Leonards-Schippers et al., 1994) or whole plant evaluations in a greenhouse, a growth chamber, and a field (Brouwer et al., 2004). Mostly leaves but also stems and tubers were evaluated for resistance (Bradshaw et al., 2006; Danan et al., 2009). High levels of field resistance to late blight are correlated with late plant maturity, which is a negative agronomic character (see the section on maturity). Therefore, in some studies, the QTL effect on resistance was corrected for the effect on maturity, resulting in QTL for maturity-corrected resistance to late blight (MCR) (Bormann et al., 2004). In total, 43 potato QTL for late blight resistance (*pLBR* loci) on all 12 chromosomes were distinguished (Table 1) between two and six per chromosome. Twenty-six of these QRL were also detected as QRL for maturity-corrected resistance to late blight. Six late blight QRL were supported by one study, another six by two studies, and thirty-one by three to fourteen studies (Figures 1–12; Supplementary Tables S1–S12). This shows a certain degree of saturation achieved in mapping potato late blight QRL. The most reproducible QRL was *pLBR5.1* on the short arm of chromosome 5 in a 6 Mbp interval, which was detected after infection with various Phytophthora races in different genetic backgrounds and geographical regions by linkage as well as association mapping (Figure 5 and Supplementary Table S5). Part but not all of the QTL effect on resistance could be explained by the QTL effect on plant maturity mapping to the same genome section (Visker et al., 2003; Bormann et al., 2004). This genome section includes the potato *R1* gene for race-specific resistance to late blight and the tomato *Bs4* gene for resistance to the bacterium *Xanthomonas campestris*. *R1* and *Bs4* have been cloned and functionally characterized. Both encode typical NBS-LRR genes (Ballvora et al., 2002; Schornack et al., 2004). The same region contains *R* genes for a virus (*Rx2* and *Nb*) and a nematode (*H2*) resistance, major QTL for nematode resistance (*Gpa, Gpa5,* and *Grp1*), a QTL for insect resistance (Colorado potato beetle = QRL *CBR5.1*), and a QTL for resistance to the fungus *Synchytrium endobioticum* (*RSe-Vb*). The syntenic tomato genome segment harbours a QTL for resistance to the fungus *Alternaria solani* (Figure 5 and Supplementary Table S5). The early observation of genetic colocalization of *R1* with a late blight QRL in potato gave rise to the hypothesis that *R* genes and QRL might have a common molecular basis before sequences of any *R* gene and whole genome sequences were known (Leonards-Schippers et al., 1994; Gebhardt and Valkonen, 2001). According to this hypothesis, the best candidate genes for the potato QRL in this 6 Mbp region are the 30 annotated disease resistance genes organized in several clusters, the largest cluster including *R1* (http://spuddb.uga.edu/jbrowse). In the syntenic tomato region, there are only 17 annotated disease resistance genes, among them is *Bs4* (https://solgenomics.net/jbrowse). This might be the reason why compared with potato, few tomato QRL map to this region.

Colocalization of *R* genes and late blight QRL was also observed on the short arm of chromosomes 4, 6, and 11 and the long arm of chromosomes 7, 8, 9.10, and 11 (Figures 4–11; Supplementary Tables S4–S11 and Supplementary Data Sheet S14). In agreement with this observation is the finding that the level of field resistance to late blight of potato varieties increased with the number of *R* genes for late blight present in these varieties (Stewart et al., 2003). Direct experimental evidence for the effect of an *R* gene on quantitative resistance to late blight was provided in the case of the sequence-characterized potato late blight resistance gene *R8* on chromosome 9, which is an NBS-LRR gene (Jiang et al., 2018). On the other hand, the number and physical size of the QRL and the fact that the potato genome contains more than 400 annotated NBS-LRR genes (The Potato Genome Sequencing Consortium, 2011) suggest that colocalizations of QRL and NBS-LRR genes may occur by chance and do not necessarily indicate a functional relationship. Moreover, there are QRL, which do not include genes annotated as disease resistance genes (NBS-LRR genes), at least not in the genome sequences used here. Examples are *pLBR1.5* and *pLBR1.6* on chromosome 1 (Figure 1 and Supplementary Table S1) or *pLBR3.3* on chromosome 3 (Figure 3 and Supplementary Table S3). The *StAOS2* locus on chromosome 11 encoded an allene oxide synthase functional in jasmonate biosynthesis and was strongly associated with MCR (Pajerowska-Mukhtar et al., 2009). When knocked down by antisense constructs in transgenic potato plants, late blight resistance was reduced, showing that this locus is functional in quantitative resistance (Pajerowska-Mukhtar et al., 2008). Therefore, genes other than *R* genes are also functional in quantitative resistance. Based on various criteria, 153 candidate genes for quantitative resistance were included in the physical chromosome maps (Supplementary Tables S1–S12), which might help in the future to identify the molecular basis of additional QRL.

In tomato, 29 late blight QRL (*tLBR* loci) were mapped on all 12 chromosomes between one and four per chromosome (Table 1; Figures 1–12, and Supplementary Tables S1–S12). Twelve QRL were supported by one study, ten by two, and seven by three to five studies (Supplementary Tables S1–S12). Most reproducible with four studies each were QRL *tLBR5.2* and *tLBR11.2* on chromosome 5 and 11, respectively, and *tLBR11.1* with five studies on chromosome 11 (Figures 5, 11 and Supplementary Tables S5, S11). QRL *tLBR9.2* and *tLBR10.2* colocalized with the tomato late blight resistance genes *Ph-3* and *Ph-2*, respectively (Table 2). *Ph-3* has been cloned and encodes an NBS-LRR gene (Zhang et al., 2014), suggesting that part of the tomato quantitative resistance to late blight is controlled by NBS-LRR genes.

Approximately half of the potato and tomato late blight QRL mapped to syntenic genomic segments (Supplementary Data Sheet S14). Colocalization by chance is to be expected, considering the number and physical size of the QRL. Nevertheless, some of these syntenic QRL might indicate that orthologous genes or gene families of tomato and potato underlie the mapped QRL.

The singular mapped pepper QTL for resistance to *P. capsici* (*Ca-qPca*) was located on the short arm of chromosome 4 and colocalized with a large cluster of potato *R* genes and QRL for late blight (Figure 4; Supplementary Table S4).

#### 3.4.2 Resistance to fungal pathogens

A variety of fungi threatens tomato and potato cultivation. To reduce the control by fungicide applications, genetic resistance is highly desirable and therefore part of breeding programs. Fourteen articles analysed and mapped QTL for resistance to five different pathogenic fungi, seven in potato, six in tomato, and one in eggplant (Supplementary Data Sheet S14 and Supplementary Table S15). Most important in potato is potato wart caused by the soil-born fungus *S. endobioticum,* which is a quarantine disease and difficult to control. Nineteen QRL for *S. endobioticum* (*RSe* loci) were physically mapped, one to two per chromosome (Table 1; Figures 1–12, and Supplementary Tables S1–S12). *Sen1* (*RSe-XIa*) on the short arm of chromosome 11 was located between 1,2 and 2.5 Mbp. It was the major quantitative wart resistance locus, which was detected in six studies. The 1,3 Mbp potato genome segment containing *Sen1* is syntenic with the inverted 0,6 Mbp tomato segment between 4,8 and 4,2 Mbp (Figure 11 and Supplementary Table S11). This segment in potato contains nineteen annotated disease resistance genes (http://spuddb.uga.edu/jbrowse), among those homologs of the tobacco *N* gene for resistance to *Tobacco Mosaic Virus* (TMV), a prototype for NBS-LRR genes (Whitham et al., 1994). Only three annotated disease resistance genes are located in the tomato syntenic region (https://solgenomics.net/jbrowse) plus the *I* gene for resistance to Fusarium wilt (*Fusarium oxysporum*). This resistance gene encodes an atypical leucine-rich-repeat receptor-like protein (LRR-RLP) (Catanzariti et al., 2017). The most distal 5 Mbp segment of the short arm of potato chromosome 11 is syntenic with the inverted tomato segment from approximately 2–5,5 Mbp. It harbours besides *Sen1* seven *R* genes (*RMc1, Rpi-cap, Ry-sto (XI), Ny-2, Na-adg, Ry-adg, and Ra-adg*) plus several QRL for resistance against different pathogens (Figure 11 and Supplementary Table S11). This region constitutes one of several ‘hotspots’ for disease resistance (Gebhardt and Valkonen, 2001) (Table 2). With two exceptions (Soltu.DM11G003310 and Soltu. DM.11G003490) all annotated disease resistance genes are located within the 1,3 Mbp region including *Sen1* and *N* homologs (http://spuddb.uga.edu/jbrowse). The *R* genes and eventually some QRL might be encoded by alleles of one or more of the nineteen NBS-LRR genes located between 1,2 and 2.5 Mbp.

**TABLE 2:** Hotspots for pathogen resistance in the potato/tomato genomes.

Chromosome	Potato segment ^a^ [Mbp]	Tomato segment ^b^ [Mbp]	Single resistance genes (bold letters) and QRL mapping to syntenic genome segments of potato/tomato	Cloned resistance genes matching the same gene or gene family in potato/tomato
chr04	0–13	0–8	***R2, Rpi-blb3, Rpi-abpt, Rpi-mcd1, Rpi-snk*** *,* ***Rpi-amr3*** *,* ***Nc-spl, Ny-tbr*** *, pLBR4.1, pCRR4.1, Gpa4,* ***Hero*** *,* ***ty-5*** *, qTy4.1, rx4.1,* ***pepy-1*** *,* ***CaPhyto*** *, and peLBR5.1*	Potato *R2, Rpi-blb3,* and *Rpi-abpt* (late blight res.); tomato *ty-5* and pepper *pepy-1* (virus res.)
chr05	0–6	1–6	***R1, H2, Nb, Rx2*** *, Gpa, Gpa5, Grp1, RSe-Va, pLBR5.1, CBR5.1,* ***Bs4*** *, SlSW-5-1, and EBR5.1*	-
chr06	0–3	2–4	***Rpi-blb2,*** *pLBR6.1, RSe-VIb,* ***Mi/Meu, Cf-2, Cf-5, Cf-6*** *,* ***Ol-4/Ol-6*** *, and* ***Pvr9***	Tomato *Mi/Meu* (nematode and insect res.), potato *Rpi-blb2* (late blight res.), and pepper *Pvr9* (virus res.)
chr07	49–55	61–66	***Gro1*** *,* ***Rpi1, Rpi2, Rpi-mch1*** *, Ts7.1, pLBR7.3,* ***I3, I1*** *, and tLBR7.2*	-
chr09	0–10	0–6	*StVe1, tLBR9.1,* ***Ve1, Ve2*** *,* ***Frl*** *, EBR9.1, WR9.1, Cm9.1, and pLBR9.1*	Tomato *Ve-1, Ve-2,* and potato *StVe1* (fungus res.)
chr09	58–67.5	62–68.5	***R9a, R8, Rpi-mcq1, Rpi-phu1, Rpi-vnt1.1, Rpi-ver1, Nx-phu, Ny-1, Ny-Smira, Ry-chc*** *, pLBR9.2, pLBR9.3, RSe-IXa, CSR9.2, Eca9, Gpa6,* ***Ph-3, Sw-5,*** *rx9.1 tLBR9.2, EBR9.2,* ***Bs3,*** *and* ***pvr6***	Tomato *Ph-3*, potato *Rpi-mcq1, R9a*, *Rpi-phu1* and *Rpi-vnt1.1* (late blight res.); tomato *Sw-5* (virus res.) and potato *R8* (late blight res.)
chr10	52–61	59–64.6	***Rpi-ber1, Rpi-ber2, Rpi-rzc1*** *, pLBR10.3, pLBR10.4, Gro1.2, RSe-Xa, Ts10.1,* ***I6,*** *Ph-2, tLBR10.2, EBR10.1, WR10.2, qTy10.1, and* ***Pvr7***	-
chr11	0–7	0–5	***Rpi-cap, Rpi-amr1, RMc1, Ry-sto XI), Ry-adg, Ra-adg, Ny-2, Na-adg*** *, pLBR11.1, GpaXI-tar, PLRV.1, EcaXIa, CSR11.1, Sen1,* ***I*** *, EBR11.1, tLBR11.1, WR11.1, and* ***N***	-
chr11	40–46.5	49–54.2	***R3a, R3b, R6/R7, R10/R11, Rm*** *, pLBR11.3, RSe-XIb,* ***I2, Rx-4/Xv3, Ty-2*** *, tLBR11.3, tLBR11.4, qTy11.1,* ***L,*** *and peCMV11.1*	Tomato *I2* (fungus res.), potato *R3a* (late blight res.), and pepper *L* (virus res.)
chr12	56–60	1–5	***Rx, Gpa2*** *,* ***MfaXIIspl,*** *pLBR12.2,* ***Mi3, Lv*** *, tLBR12.1, tCMV12.1, tBWR12.1, Ol-qtl2, EBR12.1, and* ***Me1***	Potato *Gpa2* (nematode res.) and *Rx* (virus res.)

^a^ Genome version DM v6.1.

^b^ Genome version SL4.0.

A single association mapping study located a potato QTL for resistance to *V. dahliae* (*StVe1*) on chromosome 9, using as markers homologs of the cloned tomato *Ve1* gene for resistance to the same pathogen (Simko et al., 2004). Tomato *Ve1* and *Ve2* are duplicated genes with syntenic positions on tomato chromosome 9 and encode cell surface-like receptors (Kawchuk et al., 2001), which correspond to three clustered homologs on potato chromosome 9. This is another example of the possible functional relationship between *R* genes and QRL (Figure 9 and Supplementary Table S9).

The majority of articles on resistance to pathogenic fungi in tomato deal with the mapping and cloning of *R* genes for resistance to *Cladosporium fulvum* and *F. oxysporum* (Supplementary Data Sheet S14). QTL mapping was performed for resistance to early blight (*EBR* loci) caused by *A. solani* and powdery mildew (*Ol-qtl* loci) caused by *Oidium lycopersici*. Fifteen *EBR* QRL (one to two per chromosome) were mapped to all chromosomes except chromosome 7 (Figures 1–12 and Supplementary Tables S1–S12). Eight *EBR* QRL were detected in one, six in two, and one (*EBR5.2*) in three of three studies. Resistance to powdery mildew (*O. lycopersici*) appears oligogenic as only three QRL were mapped based on three studies. *Ol-qtl1* on chromosome 6 was the only QRL detected in all three studies and colocalized with the *Ol-1* locus and *EBR6.1* (Figure 6 and Supplementary Table S6). *Ol-qtl2* and *Ol-qtl3* were both located on chromosome 12. *Ol-qtl2* colocalized with *EBR12.1* and the *Lv* gene for resistance to powdery mildew caused by *Leveillula taurica* (Figure 12 and Supplementary Table S12).

Clear synteny between potato and tomato QTL for resistance to pathogenic fungi was not observed except between *StVe1* and *EBR9.1* on chromosome 9 and between the *Sen1* locus and *EBR11.1* on chromosome 11.

#### 3.4.3 Resistance to bacterial pathogens

Thirty-eight articles report mapping and cloning of genes for resistance to bacterial pathogens (Supplementary Table S15). Twenty-four analysed and mapped QTL for resistance to five species, sixteen in tomato, seven in potato, and one in eggplant (Supplementary Data Sheet S14). Chromosome 1 carried nine QRL, the highest number of QRL for bacteria, followed by chromosomes 6 and 11 with seven QRL each.

Genetics of resistance to bacterial wilt caused by *R. solanacearum* was studied mainly in tomato (nine articles) but also in potato and eggplant (one article each). Eight QRL (*BWR* loci) were mapped in tomato, five in potato, and two in eggplant (Table 1). Most reproducible were tomato QRL *tBWR6.1* and *tBWR6.2* on chromosome 6, each detected in five studies (Figure 6 and Supplementary Table S6). The two eggplant QRL colocalized with tomato *BWR6.1* and *BWR3.1* on chromosomes 6 and 3, respectively. One of the five potato QRL for *R. solanacearum*, p*BWR11.1* on chromosome 11, could be limited to a 1,7 Mbp interval, which does not contain annotated disease resistance genes in the sequenced potato/tomato genomes (Figure 11 and Supplementary Tables S11).

Three articles located fourteen tomato QTL for resistance to *Xanthomonas* species (*rx* loci) on seven chromosomes (Table 1). The only QRL supported by all three studies was QRL *rx5.2* on chromosome 5, which colocalized with QRL *Cm5.3* and the *R* gene *Rx3* for *Xanthomonas* resistance (Figure 5; Supplementary Table S5 and Supplementary Data Sheet S14).

Ten tomato QTL for resistance to *Clavibacter michiganensis* (*Cm* loci) were mapped to eight chromosomes in four studies (Table 1). Two QRL, *Cm2.1* and *Cm5.3* on chromosomes 2 and 5 (Figures 2, 5; Supplementary Tables S2, S5), respectively, were supported by two of the four studies.

Specific for potato was a single QTL mapping experiment for resistance to *Erwinia carotovora* ssp. *atroseptica* (*Eca* loci) and five studies on QTL for resistance to *Streptomyces scabies* (common scab, *CSR* loci) (Supplementary Data Sheet S14). Nine *Eca* QRL on nine chromosomes and fifteen *CSR* QRL on ten chromosomes were distinguished (Table 1; Figures 1–12, and Supplementary Tables S1–S12). There was little consistency between the five mapping experiments on resistance to *S. scabies*. Only one locus, *CSR1.2* on chromosome 1, was supported by two studies (Figure 1 and Supplementary Table S1).

Colocalization and synteny between QTL for resistance to different pathogenic bacteria were observed in eight potato/tomato genome segments (Supplementary Data Sheet S14), which might indicate similarities between the underlying genes. Interestingly, the tomato QRL *rx5.1* and *Cm5.2* colocalized with the *Pto* and *Prf* genes for resistance *to P. syringae* (Figure 5; Supplementary Table S5 and Supplementary Data Sheet S14).

#### 3.4.4 Resistance to pathogenic viruses

A multitude of viruses infects Solanaceous crops leading to losses of crop yield and quality. In the vegetatively propagated potato, viruses are transmitted to the next tuber generation leading to progressive yield reduction, which is the reason for seed tuber production under virus-free conditions. The 70 articles on mapping and cloning of virus resistance loci in potato and tomato (Supplementary Table S15) deal mainly with monogenic resistance. Eighteen of 37 mapped virus resistance genes (20 in potato, 8 each in tomato and pepper, and 1 in tobacco) have been identified at the molecular level (7 each in tomato and pepper and 4 in potato). The highest number of virus resistance loci maps to chromosome 11 in two clusters on the short and the long arm (Figure 11 and Supplementary Table S11). Only nine articles dealt with quantitative resistance to five viruses, five in tomato, three in pepper, and one in potato (Supplementary Data Sheet S14 and Supplementary Table S15).

Six QTL for resistance to *Tomato Yellow Leaf Curl Virus* (*qTy* loci) mapped to six tomato chromosomes (Table 1; Figures 1–12, and Supplementary Tables S1–S12), three of which were supported by two studies (*qTY4.1, qTY10.1, and qTy11.1*). Two *qTy* QRL colocalized with monogenic TYLCV resistance: *qTy4.1* on chromosome 4 colocalized with the recessive resistance genes *ty-5* of tomato and *pepy-1* of pepper, both encoding the messenger RNA surveillance factor *Pelota* (Lapidot et al., 2015; Koeda et al., 2021). *qTy11.1* on chromosome 11 colocalized with the dominant resistance gene *Ty-2*, an NBS-LRR gene (Yamaguchi et al., 2018).

One QTL for resistance to *Cucumber Mosaic Virus* (*CMV* loci) was mapped in tomato and two in pepper (Table 1). The pepper QRL *peCMV11.1* colocalized with the pepper NBS-LRR gene *L* for tobamovirus resistance (Tomita et al., 2011) on the long arm of chromosome 11 (Figure 11 and Supplementary Table S11).

A major tomato QTL for resistance to *Tomato Spotted Wilt Virus* (TSWV), probably an NBS-LRR gene (Qi et al., 2022), mapped on the short arm of chromosome 5 (Figure 5 and Supplementary Table S5).

Four pepper QTL for resistance to potyviruses were localized on potato/tomato chromosomes 3, 7, 8, and 11 (Figures 3, 7, 8, 11; Supplementary Table S3, S7, S8, S11). *qPvr3.1* on chromosome 3 colocalized with the recessive potyvirus resistance genes *pvr1,2,5* from pepper and *pot-1* from tomato. Both encode the eukaryotic translation initiation factor 4E (Ruffel et al., 2002; 2005), suggesting that this QRL might be an effect of this gene.

One QTL mapping experiment in potato resistance to *Potato Leafroll Virus* (PLRV) detected a major QRL (*PLRV.1*) on chromosome 11 and a minor QRL (*PLRV.2*) on chromosome 6. *PLRV.1* was one component of a cluster of potato genes for resistance to different viruses between 0 and 5 Mbp on the short arm of chromosome 11 (Figures 6, 11; Table 2, and Supplementary Tables S6, S11).

#### 3.4.5 Resistance to nematodes

Soil parasitic nematodes infest and damage plant roots leading to yield losses. Nematode infestations are difficult to control and therefore quarantined. Twelve *R* genes for resistance to soil parasitic nematodes of the genus *Meloidogyne* and *Globodera* were mapped in potato (seven genes), tomato (four genes), and pepper (one gene), four of which were characterized at the molecular level (*Mi, Hero, Gro1-4, and Gpa2*). Twelve articles analysed quantitative resistance to nematodes in potato, mainly to the root cyst nematode *G. pallida*, which is a serious problem in potato cultivation (Supplementary Data Sheet S14 and Supplementary Table S15). Resistance to *G. pallida* was oligogenic. Only six QRL on chromosomes 4, 5, 9, and 11 were identified in ten mapping studies (Figures 4, 5, 9, 11; Supplementary Tables S4, S5, S9, S11). Three major QTL for nematode resistance were mapped reproducibly to the same potato genome segment between 4 and 7 Mbp on chromosome 5 and colocalized with the *H2* gene for resistance to *G. pallida*. One of these QRL (*Grp1*) was effective not only against *G. pallida* but also against the closely related species *G. rostochiensis* (van der Voort et al., 1998). This major nematode resistance locus constitutes part of the resistance hotspot on the short arm of potato chromosome 5 (Figure 5; Table 2, and Supplementary Table S5).

#### 3.4.6 Resistance to insects

Regarding the genetics of resistance to insect pests, the information available in the literature was limited to 11 articles, nine dealing with quantitative resistance, four in potato and five in tomato (Supplementary Data Sheet S14 and Supplementary Table S15). The most damaging insect pest in potato is the Colorado potato beetle (*Leptiotarsa decemlineata*). Based on three studies, eight QTL for resistance to Colorado beetle (*CBR* loci) were mapped to seven chromosomes (Table 1; Figures 1–12, and Supplementary Tables S1–S12). *CBR1.1, CBR5.1, CBR8.1,* and *CBR10.1* were detected in two studies based on the same mapping population. They did not overlap with the major QRL *CBR2.2* on chromosome 2 reported in the third study.

The larvae of the potato tuber moth *Tecia solanivora* are a serious threat to potato cultivation in South America. One study reported twelve QTL for resistance to *T. solanivora* (*Ts* loci) which were located on nine chromosomes (Table 1; Figures 1–12, and Supplementary Tables S1–S12).

Whiteflies transmit viruses and are controlled by insecticides, especially in greenhouses. They are particularly relevant for tomato, where greenhouse cultivation is common. Genetics of quantitative resistance to whitefly in tomato was the subject of four articles (Supplementary Data Sheet S14 and Supplementary Table S15). Ten QTL for whitefly resistance (*WR* loci) were mapped to seven chromosomes (Table 1), three of which were detected in two studies, *WR2.1, WR9.2,* and *WR10.1* on chromosomes 2, 9, and 11, respectively (Figures 2, 9, 10; Supplementary Table S2, S9, S10). A single study identified two QTL for resistance to two-spotted spider mites (*Tetranychus urticae*) on chromosome 2 (*Rtu2.1* and *Rtu2.2*).

Chromosome 2 harboured with six the most QTL for resistance to insects. Colocalization was observed between potato QRL *Ts2.1* and *CBR2.1* and between tomato QRL *Rtu2.2* and *WR2.1*. The latter two tomato QTL for insect resistance mapped syntenic with potato QRL *Ts2.2* and might be caused by the type and density of trichomes and the quantitative variation of metabolites, particularly acyl sugars (Vosman et al., 2019). Tomato QRL *WR1.1* and potato QRL *CBR1.1* also mapped to syntenic segments on chromosome 1. Tomato *Rtu2.1* and potato *CBR2.2* were syntenic on chromosome 2 and tomato *WR10.2* and potato *Ts10.1* on chromosome 10 either by chance or by identity or similarity of the underlying genes (Figures 1, 2, 10; Supplementary Tables S1, S2, S10).

#### 3.4.7 Hotspots for pathogen resistance in the Solanaceae

Pathogen resistance loci are located on all chromosomes (Figures 1–12). However, some genome segments are outstanding in harbouring numerous single genes for resistance as well as QRL for different types of pathogens. They are summarized in Table 2 and are considered hotspots for pathogen resistance in the genomes of tomato, potato, and their wild relatives, maybe also in syntenic genome segments of other Solanaceous species. Particularly striking are the clusters of *R* genes and QRL on chromosomes 9 and 11. The clustering of resistance factors of different origins and different pathogen specificity can be explained when orthologous genes from different species confer resistance either to the same pathogen or to different types of pathogens. Alternatively, clustered members of the same gene family may confer resistance to diverse pathogens. Direct experimental evidence for this concept has been provided in some cases. For example, it was shown that the tomato gene *Mi* for resistance to the nematode *M. incognita* on chromosome 6 is identical to the gene *Meu* for resistance to the potato aphid (Rossi et al., 1998; Vos et al., 1998). *Mi/Meu* is one member of a family of annotated resistance genes located between 2,3 and 2.8 Mbp on tomato chromosome 6, which is syntenic with 700 Kbp between 1,1 and 1.8 Mbp in potato. Furthermore, the sequences of the tomato gene *Mi/Meu*, the potato gene *Rpi-blb2* for late blight resistance, and the pepper gene *Pvr9* for resistance to *Pepper Mottle Virus* matched different members of the same NBS-LRR gene family (Table 2 and Supplementary Table S6). The NBS-LRR gene family in this syntenic genome segment seems to be a source of resistance factors against multiple pathogens in the Solanaceae. Other examples are the potato genes *Rx* and *Gpa2* for resistance to *Potato Virus X* (PVX) and the nematode *G. pallida*, respectively, which were shown to be members of the same clustered family of NBS-LRR genes on chromosome 12 (Van der Vossen et al., 2000). Additional examples of cloned *R* genes from different Solanaceous species matching the same gene or gene family are shown in Table 2 and Supplementary Tables S2, S4, S9, S11.

### 3.5 Physical chromosome maps of QTL for sugar content, yield, and maturity

Besides pathogen resistance, three further quantitative traits were selected for physical mapping and comparisons of QTL positions between potato and tomato, namely, the sugar content of fruits and tubers, fruit and tuber yield, and fruit or plant maturity. The sugar content of tubers and fruits is based on the same biochemical processes, that is carbohydrate metabolism. Yield and maturity of tomato fruits and potato tuber yield and plant maturity might be controlled to some extent by homologous genes. This homology might extend to other species of the Solanaceae. Colocalization and synteny of QTL might point eventually to a common molecular basis. Each mapped QTL comprised several mega base pairs of sequence including hundreds of annotated genes. More than one gene in a particular genome segment might be responsible for the observed QTL effect. It should also be kept in mind that colocalization and synteny of QTL may be by chance, and the underlying genes and mechanisms are functionally independent.

Sixty-nine publications, 45 in tomato and 24 in potato, were evaluated, which describe the genetic mapping and in very few cases the cloning of these QTL (Supplementary Table S15). The nomenclature used for the QTL varied between articles or was altogether absent. A uniform nomenclature (Table 3) was therefore adopted for QTL mapping to the same genome segment to facilitate comparisons.

**TABLE 3:** Number of physically mapped QTL for sugar content, yield, and maturity in tomato and potato.

Trait (QTL acronym)	Tomato	Potato
Tomato fruit soluble solids or sugar content (*tSS*); potato tuber sugar content, chip quality, or color (*pTSC*)	47	27
Tomato fruit yield (*tFY*); potato tuber yield (*pTY*)	30	41
Tomato fruit weight (*tFW*); potato tuber weight (*pTW*)	55	8
Tomato fruit maturity or earliness (*tM*); potato plant maturity or earliness (*pM*)	29	30

#### 3.5.1 Sugar content

The sugar content of tomato fruits and potato tubers is an important quality criterion in breeding programs of both species. The high sugar content of tomato fruits is valued for culinary quality but is negatively correlated with yield (Tanksley et al., 1996). On the contrary, tuber sugar content is considered a negative character as the accumulation of fructose and glucose during tuber storage, particularly at low temperatures, negatively affects the quality of processed potato products, such as chips and French fries. Sucrose, fructose, and glucose are products of carbohydrate metabolism, e.g., starch degradation. Many genes encoding the proteins functional in carbohydrate metabolism and transport have been biochemically and molecularly characterized in many species. They are obvious functional candidates for QTL for sugar content. One hundred and sixteen such candidate genes were included in the physical chromosome maps (Supplementary Tables S1–S12). The sugar contents of fruits and tubers of the individuals of a mapping population were measured directly in aqueous tissue extracts by enzymatic assays or indirectly: in tomato as soluble solids content based on the Brix index and in potato based on the colour of fried potato chips.

Thirty-two articles reported the QTL of tomato fruit sugar content (Supplementary Tables S15 and Supplementary Data Sheet S14). Physical mapping distinguished 47 QTL (*tSS* loci, Table 3) between two (chromosome 12) and seven (chromosome 2) QTL per chromosome (Figures 1–12 and Supplementary Tables S1–S12). The size range of the QTL was between 1 and 15 Mbp with an average of 3,6 Mbp. Thirty-five *tSS* loci were supported by three or more studies. Most reproducible were thirteen QTL (*tSS1.4, tSS1.5, tSS2.4, tSS2.5, tSS3.4, tSS4.4, tSS5.3, tSS6.3, tSS9.1, tSS9.3, tSS10.1, tSS10.3,* and *tSS12.1*), each supported by six to ten studies (Supplementary Tables S1–S12). Two-thirds of the *tSS* QTL colocalized with yield QTL, corroborating the correlation between fruit sugar content and yield. Colocalizing QTL for fruit yield and sugar content could be the result of the pleiotropic effects of the same gene(s). So far, only one tomato gene underlying a QTL for fruit sugar content was identified at the molecular level. This turned out to be one (*lin5*) of a duplicated pair of invertase genes (*lin5/lin7*) on chromosome 9, to which high-resolution linkage mapping limited the sugar QTL *Brix9-2-5* (Fridman et al., 2000). The highly reproducible QTL *tSS9.1* (seven studies) includes the invertase locus *lin5/lin7*. The orthologous potato invertase locus *INV-GE/GF* is a strong candidate for the highly reproducible QTL *pTSC9.1* for tuber sugar content (Figure 9 and Supplementary Tables S9). Recent approaches based on genome-wide association analysis (GWAS) with large numbers of SNP markers identified further candidate genes for fruit sugar QTL, without *a priori* assumptions on gene function (Tripodi et al., 2021; Zhao et al., 2022), fifteen of which were included in Supplementary Tables S1–S12.

The evaluation of eight QTL studies in potato (Supplementary Table S15 and Supplementary Data Sheet S14) resulted in 27 QTL for tuber sugar content, chip quality, or chip colour (*pTSC* loci, Table 3) in the size range from 2 to 9 Mbp with an average of 4,5 Mbp (excluding three QTL ≥15 Mbp). As in tomato, chromosome 2 carried with four QTL the highest number of sugar QTL. Due to the limited number of experiments, the reproducibility of QTL for tuber sugar content was rather low. Only four QTL were supported by three or more studies (*pTSC3.2, pTSC5.1, pTSC9.1,* and *pTSC10.2*). Among those was the most reproducible QTL *pTSC9.1* (six studies), which includes the candidate invertase locus *INV_GE/GF* (see above). Association between tuber sugar content and DNA variants within the invertase locus *INV_GE/GF* was observed besides associations of some other genes functional in carbohydrate metabolism (Li et al., 2008; Schreiber et al., 2014).

Synteny between potato *pTSC* and tomato *tSS* sugar QTL was observed for 16 of 27 *pTSC* loci (Figures 1–12; Supplementary Tables S1–S12 and Supplementary Data Sheet S14), suggesting that fruit and tuber sugar content might be partially controlled by homologous genes, for example, invertase genes.

#### 3.5.2 Yield

Tomato fruit and potato tuber yield are essential characters in breeding programs. The yield was quantified in various ways as average fruit or tuber weight per plant, per plot, or area unit. Quantitative genetics of tomato average fruit weight and total yield were most extensively studied. The integration of 42 QTL mapping experiments (Supplementary Table S15 and Supplementary Data Sheet S14) into the tomato physical map resulted in 55 QTL for fruit weight (*tFW* loci) and 30 QTL for fruit yield (*tFY* loci) (Table 3), demonstrating the complexity of this character. Excluding three QTL ≥15 Mbp, QTL intervals ranged from 0,1 to 10 Mbp with an average of 3,5 Mbp. Fruit weight and total yield are highly correlated traits (Fulton et al., 1997). The majority of fruit yield QTL colocalized with fruit weight QTL (Figures 1–12 and Supplementary Tables S1–S12), suggesting that they are largely based on the same set of genes. The highest density of tomato yield QTL was observed on chromosomes 1, 2, and 3. Forty-six QTL were supported by three or more studies. Most reproducible were QTL *tFW2.4, tFW2.5, tFW3.3,* and *tFW11.4* on chromosomes 2, 3, and 11, respectively, each of which was mapped in 12–16 studies. Yield QTL mapping to the same genome segment in independent experiments was analysed in the progeny of interspecific hybrids between cultivars and wild tomato species, with the exception of the cherry tomato *S. lycopersicum* var. *cerasiforme*. Wild tomatoes and *S. lycopersicum* var. *cerasiforme* have generally smaller fruits and lower yields compared with cultivated tomatoes. Most tomato yield QTL were therefore species unspecific. They represent rather the genomic landscape of domestication, during which fruit size and yield were increased by selection (Lin et al., 2014). Two genes underlying tomato yield QTL have been identified at the molecular level. The highly reproducible QTL *tFW2.5* on chromosome 2 (13 studies) colocalized with *tFY2.3* (Figure 2 and Supplementary Table S2) and includes *fw2.2*, the first gene identified at the molecular level underlying a yield QTL (Frary et al., 2000). Colocalizing QTL *tFW3.3* (14 studies) and *tFY3.2* on chromosome 3 include a cytochrome P450 gene that controls fruit weight (Chakrabarti et al., 2013). The potato QTL *pTY3.2* maps to the syntenic region (Figure 3; Supplementary Tables S3 and Supplementary Data Sheet S14). Natural variation in hundreds of genes in most functional categories has probably a direct or indirect effect on yield. Among them are genes functional in central metabolism such as glycolysis, Calvin cycle, tricarboxylic acid (TCA) cycle, and other pathways. Seventy-six characterized genes of this type were included in the physical maps (Supplementary Tables S1–S12). GWAS identified 37 novel candidate genes for fruit weight QTL (Sacco et al., 2015; Tripodi et al., 2021; Zhao et al., 2022), which are also included in Supplementary Tables S1–S12.

Compared with tomato, few QTL mapping experiments addressed potato tuber yield. The evaluation of 10 articles (Supplementary Table S15 and Supplementary Data Sheet S14) resulted in 41 QTL for tuber yield (*pTY* loci) and eight for tuber weight (*pTW* loci) (Table 3). Excluding six QTL ≥15 Mbp, QTL intervals ranged from 1,6 to 13 Mbp with an average of 4,7 Mbp. Like in tomato, most tuber weight QTL colocalized with tuber yield QTL. Ten yield QTL (*pTY2.2, pTY2.3, pTY2.4, pTY3.1, pTY5.1, pTY5.2, pTY6.2, pTY7.2, pTY9.3,* and *pTY12.1*) were supported by three or more studies (Figures 1–12; Supplementary Tables S1–S12). The QTL *pTY2.2* on chromosome 2 and *pTY5.1* on chromosome 5 were most reproducible with five and seven supporting studies, respectively (Figures 2, 5; Supplementary Tables S2, S5). Genes controlling tuber yield QTL have not been formally cloned and characterized. However, candidate genes for yield QTL are available and were included in the physical maps. They resulted from the pairwise comparison of potato genotype pools with differential tuber yield with the 8,3 k SolCap SNP array. SNPs with differential allele frequency between the genotype pools were identified, some of which resulted in amino acid changes in annotated genes (Schönhals et al., 2017). One hundred and ninety-four genes of this type were included as candidate genes in the physical maps (Supplementary Tables S1–S12). None of them were identical to the novel candidate genes for tomato fruit weight identified by GWAS. As for tomato fruit yield, the 67 genes functional in central metabolism are also candidate genes for potato tuber yield. The large and certainly incomplete number of candidate genes combined with the number and genome coverage of yield QTL resulted in multiple colocalizations of QTL and candidate genes. Further functional analysis is required to verify a possible causal relationship between a QTL and natural allelic variation at candidate gene loci.

Twenty-eight yield QTL (*tFW, tFY, pTY,* and *pTW* loci) appeared syntenic in tomato and potato (Figures 1–12; Supplementary Tables S1–S12 and Supplementary Data Sheet S14). Due to the presence of yield QTL on every chromosome arm in both tomato and potato, the assumption that synteny is observed because orthologous genes control a syntenic QTL might not be justified in many cases. Nevertheless, the observation that chromosomes 1 and 2 of both tomato and potato are particularly rich in yield QTL suggests that the molecular basis of some yield QTL might be related.

#### 3.5.3 Maturity

The maturity or earliness of fruits or plants is an agronomic character in annual crops that is relevant for agricultural practice, where early maturity is preferred. In tomato, the trait describes the status of fruit ripening, and in potato, the trait describes the vegetative period from plant emergence, growth, flowering, and tuberization to senescence. Early maturity is correlated with lower yields in tomato and potato (Maris, 1969; Bernacchi et al., 1998). Moreover, earlier maturity in potato is correlated with higher susceptibility to late blight (Collins et al., 1999; Visker et al., 2003; Bormann et al., 2004). High-yielding varieties with acceptable earliness and high field resistance to late blight are highly desirable breeding goals. Maturity or earliness was visually scored in various ways and recorded in numerical scales from 1 to 5 or 1 to 9. The trait was the subject of twelve QTL mapping experiments in potato and eight in tomato (Supplementary Table S15 and Supplementary Data Sheet S14).

Maturity or earliness was evaluated together with late blight resistance in nine of 12 potato QTL studies. In total, 30 QTL were mapped (*pM* loci) (Table 3), of which 23 overlapped with late blight QRL. Excluding two QTL ≥15 Mbp, QTL size ranged from 1 to 12 Mbp with an average of 4,5 Mbp. Only two maturity QTL were reported in three or more studies (*pM3.1 and pM5.1*). The most reproducible QTL was *pM5.1* on the short arm of chromosome 5 (eight studies) (Figure 5 and Supplementary Table S5). Plant maturity in potato depends on the adaptation of tuberization to the daylength in northern latitudes. Here, the crop is grown under long-day conditions, whereas in its original habitats in central South America, the potato’s life cycle is adapted to short days. Short-day adapted genotypes initiate tubers and mature very late under long-day conditions. The gene *StCDF1* encoding a DOF (DNA-binding with one finger) transcription factor has been identified, which controls all or part of the effect of the major QTL *pM5.1* (Kloosterman et al., 2013). The tuberization-control gene *StSP6A*, a paralogue of the Arabidopsis flowering locus T (*FT*) (Navarro et al., 2011), is located on the long arm of chromosome 5 within the range of maturity QTL *pM5.2.* It is a functional candidate gene for this QTL.

Twenty-nine QTL for fruit maturity (*tM* loci) were distinguished in tomato (Table 3), in a size range from 1 to 12 Mbp with an average of 3,6 Mbp. Six maturity QTL were supported by three or more studies (*tM1.1, tM2.3, tM5.2, tM5.3, tM9.1,* and *tM11.2*). Most reproducible with five studies each were *tM2.3* and *tM5.3*. The tomato orthologue of *StSP6A* might be a candidate gene for QTL *tM5.3* (Supplementary Data Sheet S14). Three-quarters of the maturity QTL overlapped with yield QTL in accordance with the correlation between these traits, suggesting that genes controlling fruit maturity may have pleiotropic effects on yield and *vice versa* (Figures 1–12; Supplementary Tables S1–S12 and Supplementary Data Sheet S14). Synteny between tomato and potato maturity QTL was observed in 13 genome segments (Supplementary Data Sheet S14).

## 4 Conclusion

The structural comparison between the tomato and potato genomes based on the physical mapping of 2,741 sequences to updated reference genome sequences detected novel intrachromosomal inversions and translocations between the otherwise collinear tomato and potato genomes.

One hundred and twelve single loci for pathogen resistance of potato, tomato, and pepper were integrated into physical maps of the 12 potato/tomato chromosomes. They have been located previously on molecular linkage maps. Linkage mapping with DNA-based markers has led to the map-based cloning and characterization of 56 resistance genes, thus making a significant contribution to the knowledge of the structure and function of plant genes for pathogen resistance. Based on the sequences of cloned resistance genes or markers tightly linked with major effect resistance loci, diagnostic markers have been developed, which have enriched the breeder’s toolbox for the selection of resistant cultivars (Tiwari et al., 2022; Marhadour and Prodhomme, 2023).

Two hundred and thirty QTL for pathogen resistance were positioned on the physical tomato/potato maps, part of which colocalized with *R* genes for resistance to the same or different pathogens. This and in very few cases the identification of the underlying gene suggest that qualitative and quantitative resistance is at least in part based on the same genes, namely, NBS-LRR type gene families. On the other hand, not all QRL can be explained by the action of *R* genes. Certain genome segments exhibited a high density of qualitative and quantitative resistance loci, so-called hotspots for pathogen resistance.

Two hundred and sixty-seven QTL for tomato fruit and potato tuber sugar content, yield, and maturity were distinguished on the physical tomato/potato chromosome maps. This is certainly an underestimation of the number of genes involved in these complex traits. Numerous colocalizations of QTL corroborate the phenotypic correlations and suggest that some genes underlying QTL for sugar content or maturity have pleiotropic effects on yield or pathogen resistance or *vice versa*.

Map-based cloning of genes underlying QTL in the Solanaceae was rarely undertaken. Only about half a dozen cases were reported in the literature. The molecular basis of QTL in the Solanaceae is still largely unknown. The main objective of QTL mapping in the past has been the identification of diagnostic markers to be used for the marker-assisted selection of superior cultivars in breeding programs. This has not materialized in applied breeding programs. Instead of genetic dissection of individual QTL, the method of choice is now genomic selection (GS), which does not require knowledge of individual QTL map positions (Meuwissen et al., 2001; Duangjit et al., 2016; Sverrisdóttir et al., 2018; Budhlakoti et al., 2022). GS is made possible by technical advances in DNA sequencing and data analysis, which make genotyping by sequencing large populations of cultivars manageable and affordable.
