# Supplementary material for: A physical map of traits of agronomic importance based on potato and tomato genome sequences
Source: Front Genet. 2023 Jul 25;14:1197206. doi: 10.3389/fgene.2023.1197206 (PMC10411547; doi:10.3389/fgene.2023.1197206)
Supplement: Supplementary file 8 [file Table13.DOCX]

**Table S13**. Cultivated and wild *Solanum* species involved in genetic mapping of traits in tomato and potato.

|  | Potato *Solanum* species | Tomato *Solanum* species |
| --- | --- | --- |
| Cultivated | *S. tuberosum* Group *tuberosum* | *S. lycopersicum*  (former *Lycopersicon esculentum*) |
|  | *S. tuberosum* Group *phureja* | *S. lycopersicum* var. *cerasiforme* |
|  | *S. tuberosum* Group *andigenum* |  |
| Wild | *S. acaule* | *S. cheesmanii* |
|  | *S. americanum* | *S. chilense* |
|  | *S. berthaultii* | *S. chmielewskii* |
|  | *S. bulbocastanum* | *S. galapagense* |
|  | *S. capsicibaccatum* | *S. habrochaites (L. hirsutum)* |
|  | *S. chacoense* | *S. parviflorum (S. neorickii)* |
|  | *S. demissum* | *S. pennellii* |
|  | *S. edinense* | *S. peruvianum* |
|  | *S. gourlayi* | *S. pimpinellifolium* |
|  | *S. michoacanum* |  |
|  | *S. microdontum* |  |
|  | *S. mochiquense* |  |
|  | *S. multidissectum* |  |
|  | *S. pampasense* |  |
|  | *S. papita* |  |
|  | *S. paucissectum* |  |
|  | *S. pinnatisectum* |  |
|  | *S. ruiz-ceballosii* |  |
|  | *S. schenckii* |  |
|  | *S. spegazzinii* |  |
|  | *S. sparsipilum* |  |
|  | *S. stenotomum* |  |
|  | *S. stoloniferum* |  |
|  | *S. tarijense* |  |
|  | *S. venturii* |  |
|  | *S. vernei* |  |
|  | *S. verrucosum* |  |
