# Supplementary material for: A physical map of traits of agronomic importance based on potato and tomato genome sequences
Source: Front Genet. 2023 Jul 25;14:1197206. doi: 10.3389/fgene.2023.1197206 (PMC10411547; doi:10.3389/fgene.2023.1197206)
Supplement: Supplementary file 14 [file Table14.docx]

**Table S14**: Number of articles evaluated, describing genetic mapping and cloning of qualitative and quantitative trait loci for pathogen resistance in potato, tomato, pepper, eggplant and tobacco.

| Pathogen class | Pathogen | Potato | Tomato | Pepper | Eggplant | Tobacco |
| --- | --- | --- | --- | --- | --- | --- |
| Oomycetes | *Phytophthora infestans, P. capsici,* late blight | 33 / 40 ^1^ | 12 / 2 | 2 / 1 |  |  |
| Fungi | *Alternaria solani* (early blight) |  | 3 / 2 |  |  |  |
|  | *Cladosporium fulvum* (leaf mould) |  | 0 / 11 |  |  |  |
|  | *Fusarium oxysporum* (wilt) |  | 0 / 12 |  | 1 / 0 |  |
|  | *Leveillula taurica* (powdery mildew) |  | 0 / 2 |  |  |  |
|  | *Oidium lycopersicum, O. neolycopersici,* (powdery mildew) |  | 3 / 6 |  |  |  |
|  | *Pyrenochaeta lycopersici* (corky root rot) |  | 0 / 1 |  |  |  |
|  | *Stemphylium* spp (leaf spot) |  | 0 / 3 |  |  |  |
|  | *Synchytrium endobioticum* (wart) | 6 / 2 |  |  |  |  |
|  | *Verticillium dahliae* (wilt) | 1 / 0 | 0 / 3 |  |  |  |
| Bacteria | *Clavibacter michiganensis* (bacterial canker) |  | 4 / 0 |  |  |  |
|  | *Erwinia (Pectobacterium) carotovora subsp. atroseptica* (blackleg, soft rot) | 1 / 0 |  |  |  |  |
|  | *Pseudomonas syringae, P. solanacearum* (bacterial speck) |  | 0 / 3 |  |  |  |
|  | *Ralstonia solanacearum* (bacterial wilt) | 1 / 0 | 9 / 0 |  | 1 / 0 |  |
|  | *Streptomyces scabies* (common scab) | 5 / 0 |  |  |  |  |
|  | *Xanthomonas campestris, X. perforans* (bacterial spot) |  | 3 / 7 | 0 / 2 |  |  |
| Viruses | *Tomato Yellow Leaf Curl Virus* (TYLCV), Begomovirus |  | 3 / 8 | 0 / 2 |  |  |
|  | *Cucumber Mosaic Virus* (CMV) |  | 1 / 0 | 2 / 0 |  |  |
|  | *Potato Leafroll Virus* (PLRV) | 1 / 1 |  |  |  |  |
|  | *Potato Virus M* (PVM) | 0 / 1 |  |  |  |  |
|  | *Potato Virus S* (PVS) | 0 / 1 |  |  |  |  |
|  | *Potato Virus X* (PVX) | 0 / 7 |  |  |  |  |
|  | *Potato Virus Y* (PVY), Potyvirus | 0 / 20 | 0 / 2 | 1 / 8 |  |  |
|  | *Tobacco Mosaic Virus* (TMV), tobamovirus |  | 0 / 4 | 0 / 3 |  | 0 / 1 |
|  | *Tomato Spotted Wilt Virus* (TSWV), tospovirus |  | 1 / 3 |  |  |  |
| Nematodes | *Globodera pallida* | 10 / 4 |  |  |  |  |
|  | *Globodera rostochiensis* | 3 / 10 | 0 / 2 |  |  |  |
|  | *Meloidogyne incognita, M. chitwodii, M. fallax* | 0 / 4 | 0 / 10 | 0/2 |  |  |
| Insects | *Bemisia tabaci, Trialeurodes vaporarium* (whitefly) |  | 4 / 0 |  |  |  |
|  | *Leptiotarsa decemlineata* (Colorado beetle) | 3 / 0 |  |  |  |  |
|  | *Macrosiphum euphorbiae* (potato aphid) |  | 0 / 2 |  |  |  |
|  | *Tecia solanivora* (potato tuber moth) | 1 / 0 |  |  |  |  |
|  | *Tetranychus urticae* (two-spotted spider mite) |  | 1 / 0 |  |  |  |

^1^ The left number is the number of articles dealing with quantitative resistance, the right number is the number of articles dealing with qualitative resistance

Table S16. Putative colocalization within and synteny between potato and tomato of QTL and *R* genes for resistance to late blight.

| Chromosome | Potato segment  [Mbp] ^1^ | Tomato segment  [Mbp] ^2^ | Colocalization within and synteny between potato/tomato of late blight QRL (*pLBR, tLBR*) and *R* genes (bold letters) |
| --- | --- | --- | --- |
| chr01 | 72 - 76 | 76 - 80 | *pLBR1.4 / tLBR1.2* |
| chr01 | 81 - 84,  87 - 88,3 | 85 – 90,6 | *pLBR1.5 and pLBR1.6/tLBR1.3* |
| chr02 | 19 - 25 | 24 - 35 | *pLBR2.2 / tLBR2.2* |
| chr03 | 0 – 6, 47 - 49 | 0 - 4 | *pLBR3.1 and pLBR3.3 / tLBR3.1* |
| chr04 | 0 - 10 | - | *pLBR4.1,* ***Rpi-blb3, R2, Rpi-abpt, Rpi-mcd1, Rpi-snk*** and ***Rpi-amr3i*** |
| chr04 | 61 - 66 | 55 - 63 | *pLBR4.2 / tLBR4.2* |
| chr05 | 0 - 6 | - | *pLBR5.1* and ***R1*** |
| chr05 | 52 – 55,2 | 63 - 64 | *pLBR5.2 / tLBR5.2* |
| chr06 | 0 - 3 | - | *pLBR6.1* and ***Rpi-blb2*** |
| chr06 | 54 - 58 | 41 – 47,2 | *pLBR6.4 / tLBR6.2* |
| chr07 | 0 - 5 | 1 - 4 | *pLBR7.1 / tLBR7.1* |
| chr07 | 50 - 54 | 61 - 66 | *pLBR7.3,* ***Rpi-mch1, Rpi1*** and ***Rpi2*** */ tLBR7.2* |
| chr08 | 44 - 52 | - | *pLBR8.2* and ***Rpi-blb1*** |
| chr08 | 53 – 59,1 | 60 – 63,9 | *pLBR8.3 / tLBR8.2* |
| chr09 | 0 - 4 | 1 - 6 | *pLBR9.1 / tLBR9.1* |
| chr09 | 58 - 63 | - | *pLBR9.2* and ***Rpi-ver1*** |
| chr09 | 64 - 67,5 | 66 - 68 | *pLBR9.3,* ***Rpi-mcq1, R9a, R8, Rpi-phu1*** and ***Rpi-vnt1.1*** */ tLBR9.2* and ***Ph-3*** |
| chr10 | 0 - 4 | 1 - 5 | *pLBR10.1 / tLBR10.1* |
| chr10 | 52 - 56, 57 - 61 | 59 – 64,6 | *pLBR10.3, pLBR10.4,* ***Rpi-ber1, Rpi-ber2*** and ***Rpi-rzc1 /*** *tLBR10.2* and ***Ph-2*** |
| chr11 | 0 - 4 | 0 - 4 | *pLBR11.1* and ***Rpi-cap /*** *tLBR11.1* |
| chr11 | 40 - 46,5 | 49 - 50,  53 – 54,2 | *pLBR11.3,* ***R3a, R3b, R6, R7, R10, R11*** and ***Rpi-smira1*** */ tLBR11.3, tLBR11.4* |
| chr12 | 56 – 59,6 | 0 - 3 | *pLBR12.2 / tLBR12.1* |

^1^ Genome version DM v6.1., ^2^ Genome version SL4.0

Table S17. Putative colocalization within and synteny between potato and tomato of QTL and *R* genes for resistance to bacterial pathogens.

| Chromosome | Potato segment  [Mbp] ^1^ | Tomato segment  [Mbp] ^2^ | Colocalization within and synteny between potato/tomato of QRL and *R* genes (bold letters) for bacterial pathogens |
| --- | --- | --- | --- |
| chr02 | 42 - 46 | 49 - 50 | *Eca2B / Cm2.1* |
| chr04 | 2 - 3 | 0 - 1 | *CSR4.1 / rx4.1* |
| chr04 | 66 - 69 | 62 - 63 | *Eca4B / rx4.2* |
| chr05 | - | 5 - 8 | ***Pto, Prf,*** *rx5.1, Cm5.2* |
| chr05 | - | 58 - 63 | *rx5.2, Cm5.3,* ***Rx3*** |
| chr06 | - | 29 - 35 | *rx6.1, BWR6.1* |
| chr06 | 45 - 47 | 37 - 40 | *CSR6.1 / BWR6.2* |
| chr09 | 59 - 63 | - | *CSR9.2, Eca9* |

^1^ Genome version DM v6.1., ^2^ Genome version SL4.0

Table S18: Number of mapping and cloning studies of QTL for sugar content, yield and maturity in potato and tomato.

| Quantitative trait measured | Tomato | Potato |
| --- | --- | --- |
| Sugar content: tomato fruit sugar content, soluble solids content, Brix index; potato tuber sugar content, chip quality or color | 32 | 8 |
| Yield: Tomato fruit yield, fruit weight, fruit size; potato tuber yield, tuber weight | 42 | 10 |
| Maturity: Tomato fruit maturity or earliness; potato plant maturity or earliness | 8 | 12 |

Table S19. Putative colocalization and synteny of QTL for yield, fruit or tuber sugar content and maturity in potato and tomato.

| Chromosome | Tomato segment  [Mbp] ^1^ | Potato segment  [Mbp] ^2^ | Colocalization within and synteny between tomato / potato QTL for yield, fruit or tuber sugar content and maturity |
| --- | --- | --- | --- |
| chr01 | 0 - 5 | 0 - 4 | *tFW1.1, tFY1.1, tSS1.1, tM1.1 / pTY1.1, pM1.1* |
| chr01 | 63 - 70 | 54 - 66 | *tFW1.2 / pTY1.2, pM1.3* |
| chr01 | 71 - 75 | - | *tFW1.3, tFY1.2, tSS1.3, tM1.2* |
| chr01 | 78 - 82 | 72 - 78 | *tFW1.4, tFY1.3, / pTY1.3, pTW1.1* |
| chr01 | 83 - 85 | - | *tFW1.5, tSS1.4* |
| chr01 | 88 - 90,6 | 84 - 88,6 | *tFW1.4, tFW1.6, tFY1.4, tSS1.5 /pTY1.4, pTSC1.1* |
| chr02 | 21 - 30 | 10 - 19 | *tFW2.1, tFY2.1, tSS2.1, tM2.1 / pTY2.1, pTSC2.1, pM2.1* |
| chr02 | 35 - 41 | 27 - 31 | *tSS2.3, tM2.2 /pM2.2* |
| chr02 | 41 - 44 | 31 - 35 | *tFW2.3, tFY2.2, tSS2.7 / pTY2.3* |
| chr02 | 44 - 47 | 36 - 40 | *tFW2.4, tSS2.4 / pTY2.4* |
| chr02 | 46 – 53,2 | - | *tFW2.5, tFY2.3, tSS2.5,* ***fw2.2*** |
| chr02 | 51 – 53,2 | 40 - 46 | *tFW2.6, tFY2.3, tSS2.6 / pTY2.5, pTSC2.2, pTM2.3* |
| chr03 | 0 - 3 | - | *tFW3.1, tFY3.1, tSS3.1, tM3.1* |
| chr03 | 13 - 31 | - | *tFW3.2, tSS3.3, tM3.2* |
| chr03 | - | 31 - 50 | *pTY3.1, pTSC3.3, pM3.1* |
| chr03 | 50 - 61 | *-* | *tFW3.3, tFW3.5, tFY3.2,* ***SlKLUH,*** *tSS3.4, tM3.3* |
| chr03 | 60 - 65 | 51 - 59 | *tFW3.4, tFY3.3, tSS3.5, tM3.4 / pTY3.2, pTSC3.4, pM3.2* |
| chr04 | 0 - 3 | 0 - 7 | *tFY4.1, tSS4.1 / pTY4.1, pTSC4.1, pM4.1* |
| chr04 | 58 - 62 | - | *tFW4.3, tSS4.3* |
| chr04 | 63 – 64,4 | 61 - 69 | *tFW4.4, tSS4.4 / pTY4.3, pTW4.1, pM4.3* |
| chr05 | 1 - 6 | 0 - 6 | *tFW5.1, tSS5.1, tM5.1 / pTY5.1, pTW5.1, pTSC5.1, pM5.1,* ***StCDF1*** |
| chr05 | - | 8 – 12 | *pTY5.2, pTSC5.2* |
| chr05 | 59 - 63 | - | *tFW5.2, tFY5.1, tSS5.2, tM5.2* |
| chr05 | 62 - 64 | 50 -55,2 | *tFW5.3, tFY5.2, tSS5.3, tM5.3 / pTY5.3, pTSC5.3, pM5.2,* ***StSP6A*** |
| chr06 | 0 - 3 | 0 - 9 | *tFW6.1, tSS6.1 / pTY6.1, pTY6.2, pTW6.1, pTSC6.1, pM6.1* |
| chr06 | 34 - 42 | - | *tFW6.3, tFY6.1, tSS6.4, tM6.1* |
| chr06 | 40 - 46 | 52 - 56 | *tSS6.3 / pTSC6.2* |
| chr06 | 45 – 47,2 | 57 – 59,4 | *tFW6.4, tFY6.2 / pTY6.3* |
| chr07 | 0 - 6 | 0 - 6 | *tFW7.1, tFY7.1, tSS7.1, tM7.1 / pTY7.1, pTSC7.1, pM7.1* |
| chr07 | 58 - 64 | 47 - 53 | *tFW7.3, tFY7.2, tSS7.2, tM7.3 / pTY7.3* |
| chr07 | 64 - 66 | - | *tFY7.3, tFW7.4, tSS7.3, tM7.3* |
| chr07 | - | 54 – 57,5 | *pTY7.4, pM7.4* |
| chr08 | 0 - 7 | 1 - 12 | *tFW8.1, tFY8.1, tSS8.1, tM8.1 / pTY8.1, pM8.1* |
| chr08 | 40 - 58 | 39 - 52 | *tFW8.2, tFY8.2, tSS8.2, tM8.2 / pTY8.2, pTSC8.1* |
| chr08 | 58 - 63,9 | 52 - 59 | *tFW8.3, tFY8.3, tSS8.3, tSS8.4, tM8,3 / pTY8.3, pTSC8.2* |
| chr09 | 0 - 5 | 0 - 5 | *tFW9.1, tFY9.1, tSS9.1,* ***lin5,*** *tM9.1 / pTW9.1, pTY9.1, pTSC9.1, pM9.1* |
| chr09 | 50 - 58 | - | *tFW9.4, tSS9.2* |
| chr09 | 60 - 65 | - | *tFW9.2, tM9.2* |
| chr09 | 63 - 68,5 | 62 - 67,5 | *tFW9.3, tSS9.3, tM9.3 / pTY9.3, pTSC9.2* |
| chr10 | 0 - 2 | 0 - 3 | *tFW10.1, tFY10.1, tSS10.1, tM10.1 / pTY10.1* |
| chr10 | 30 - 34 | - | *tFY10.2. tM10.2* |
| chr10 | 50 - 62 | 58 - 61 | *tFW10.3, tFY10.3, tSS10.3 / pTY10.3, pM10.1* |
| chr10 | 63 - 65 | 51 - 57 | *tFY10.4, tSS10.4 / pTY10.2, pTSC10.1* |
| chr11 | 0 - 5 | 0 - 6 | *tFW11.1, tSS11.1, tM11.1 / pTY11.1, pTY11.2, pM11.1* |
| chr11 | - | 7 -11 | *pTY11.3, pTSC11.1* |
| chr11 | 30 - 38 | 30 - 34 | *tFW11.3, tM11.2 / pM11.2* |
| chr11 | - | 38 - 45 | *pTY11.5, pTW11.1, pM11.3* |
| chr11 | 52 – 54,2 | 44 – 46,5 | *tFW11.4, tSS11.3 / pTSC11.2* |
| chr12 | 1 - 7 | 57 – 59,6 | *tFY12.1, tFW12.1, tFW12.2, tFY12.2, tSS12.1, tM12.1 / pTY12.3, pM12.3* |
| chr12 | 61 – 66,6 | 0 - 9 | *tFW12.3, tFY12.3, tSS12.2, tM12.2, tM12.3 / pTY12.1, pTW12.1, pM12.1* |

^1^ Genome version SL4.0, ^2^ Genome version DM v6.1.
